# Efficient Unnatural Protein Production by Pyrrolysyl-tRNA Synthetase With Genetically Fused Solubility Tags

**DOI:** 10.3389/fbioe.2021.807438

**Published:** 2021-12-23

**Authors:** Nikolaj G. Koch, Tobias Baumann, Nediljko Budisa

**Affiliations:** ^1^ Biokatalyse, Institut für Chemie, Technische Universität Berlin, Berlin, Germany; ^2^ Bioanalytik, Institut für Biotechnologie, Technische Universität Berlin, Berlin, Germany; ^3^ Chemical Synthetic Biology, Department of Chemistry, University of Manitoba, Winnipeg, MB, Canada

**Keywords:** genetic code expansion, pyrrolysyl-tRNA synthetase, non-canonical amino acid, protein engineering, solubility tags, stop codon suppression, *S*-allyl-l-cysteine, aminoacyl-tRNA synthetase

## Abstract

Introducing non-canonical amino acids (ncAAs) by engineered orthogonal pairs of aminoacyl-tRNA synthetases and tRNAs has proven to be a highly useful tool for the expansion of the genetic code. Pyrrolysyl-tRNA synthetase (PylRS) from methanogenic archaeal and bacterial species is particularly attractive due to its natural orthogonal reactivity in bacterial and eukaryotic cells. However, the scope of such a reprogrammed translation is often limited, due to low yields of chemically modified target protein. This can be the result of substrate specificity engineering, which decreases the aminoacyl-tRNA synthetase stability and reduces the abundance of active enzyme. We show that the solubility and folding of these engineered enzymes can become a bottleneck for the production of ncAA-containing proteins *in vivo*. Solubility tags derived from various species provide a strategy to remedy this issue. We find the N-terminal fusion of the small metal binding protein from *Nitrosomonas europaea* to the PylRS sequence to improve enzyme solubility and to boost orthogonal translation efficiency. Our strategy enhances the production of site-specifically labelled proteins with a variety of engineered PylRS variants by 200–540%, and further allows triple labeling. Even the wild-type enzyme gains up to 245% efficiency for established ncAA substrates.

## Introduction

Genetic code expansion (GCE) has proven to be a major tool for adding new chemistries to the biological world (Liu and Schultz, 2010; Chin, 2017; Mukai et al., 2017). In this regard, amino acids with non-proteinogenic functional groups can be used to elucidate enzyme functions (Groff et al., 2009; Minnihan et al., 2011; Baumann et al., 2019), to tailor protein properties (Agostini et al., 2017; Li et al., 2019) or to encode proteins with functions not known to nature (Drienovská et al., 2018; Burke et al., 2019). A straightforward strategy to achieve this functionalization is to engineer aminoacyl tRNA synthetase (aaRS)/tRNA pairs, which enable the ribosomal incorporation of non-canonical amino acids (ncAAs) in response to a reprogrammed codon. These aaRS/tRNA pairs form the essential parts of orthogonal translation systems (OTSs). The most commonly used approach for this site-specific peptide and protein modification is stop codon suppression (often targeting the amber stop codon). Here, the ncAA is incorporated in response to an in-frame stop codon placed at a predefined position in the protein coding sequence, ribosomally expressed either *in vivo* or *in vitro* (Liu and Schultz, 2010; Chin, 2017; Mukai et al., 2017).

The majority of all engineered aaRS variants to date are derived from the *Methanosarcina mazei/barkeri* pyrrolysyl-tRNA synthetases (*Mm*PylRS/*Mb*PylRS) or the *Methanocaldococcus jannaschii* tyrosyl-tRNA synthetase (*Mj*TyrRS). The archaeal origin and therefore distant phylogeny constitute the reason for their orthogonality in bacterial cells and consequential easy implementation as OTSs. PylRS/tRNA^Pyl^ pairs from the species of *Methanosarcina* are a particularly attractive choice for GCE due to their orthogonal reactivity in both bacterial and eukaryotic cells (Liu and Schultz, 2010; Chin, 2017; Mukai et al., 2017). Since its discovery, the PylRS system has been engineered to incorporate over 100 ncAAs (Wan et al., 2014; Dumas et al., 2015). In the beginning, mainly longer aliphatic pyrrolysine (Pyl) analogs and later also Phe and His analogs could be inserted into recombinant proteins (Wang et al., 2012; Xiao et al., 2014). Recently, this has been significantly extended to Trp analogs (Tseng et al., 2020) and small aliphatic side chain containing ncAAs (Koch et al., 2021).

PylRS-based OTSs now enable the incorporation of all types of side chains into proteins: Small/short, large, bulky, long aliphatic, small-aliphatic, aromatic, polar, polarizable, ionizable, neutral, hydrophobic, hydrophilic including various bioorthogonal chemical functionalities along rarely used elements (fluorine or boron) and chemical entities (Baumann et al., 2016). The variety of examples indicate that the PylRS binding pocket can be engineered to accommodate almost every substrate and therefore opens up opportunities to focus on the remaining obstacles in the field of GCE. One of these is the low efficiency of ribosomal ncAA incorporation caused by the comparatively low aaRS enzyme activity, ultimately leading to low yields of target protein (O’Donoghue et al., 2013; Exner et al., 2017).

On the contrary, OTSs based on other aaRS scaffolds can already produce target proteins at wild-type level (Hauf et al., 2017; Schwark et al., 2018). To some extent, the low efficiency can be compensated by supplying more ncAA to the growth medium. The standard is 1 mM, but often higher concentrations of up to 10 mM of in-house synthesized or purchased ncAA are used, pushing the cost of unnatural protein production up (Liu et al., 2018; Willis and Chin, 2018; Galindo Casas et al., 2020). Many efforts have been made to progressively increase protein production. These include optimization of the OTS plasmid copy number and promotor strength of the aaRS and/or tRNA genes (Chatterjee et al., 2013), engineering tRNA^Pyl^ (Fan et al., 2015), directed evolution of the aaRS (Bryson et al., 2017), optimization of the sequence context surrounding the target codon (Pott et al., 2014), host cell engineering (Lajoie et al., 2013) and engineering parts of the translational machinery (e.g., elongation factor TU) (Park et al., 2011). All these strategies have been effective in increasing the ncAA containing target protein yield, which, however, still remains below the levels of recombinant production without concurrent ncAA modification.

Enabling the incorporation of new ncAAs commonly requires mutations in the aaRS active site. Since most mutations are destabilizing, this can have substantial effects on the overall protein stability, leading to a lower abundance of correctly folded enzyme and promoting protein aggregation (Tokuriki et al., 2008a; Tokuriki and Tawfik, 2009). With the aforementioned alterations impacting enzyme stability, it has been proposed that aaRS scaffolds with high thermostability provide better starting points for enzyme engineering efforts (Grasso et al., 2021). Unfortunately, most thermophilic enzymes with the necessary stability to tolerate these mutations experience low catalytic activity at standard cultivation conditions for commonly used hosts cells (e.g., *E. coli*) (Sarmiento et al., 2015). Understandably, the majority of application-driven aaRS engineering efforts have been directed towards substrate specificity—to allow incorporation of new ncAAs and genetic encoding of chemical functionalities. Surprisingly, there are no studies in which the physico-chemical properties of PylRS are rationally modified, to determine if poor protein folding and low solubility are major bottlenecks for OTS efficiency *in vivo*. A classical approach to increase the production of soluble and active recombinant proteins is to genetically fuse a well-folding, highly soluble protein domain to the N- or C-terminus of the target (Esposito and Chatterjee, 2006; Zhou and Wagner, 2010; Paraskevopoulou and Falcone, 2018). To our knowledge, this approach has not been tested for improving PylRS-based OTS performance.

Besides serving as our model substrate, the attractiveness for Sac incorporation into peptides and proteins stems from the introduction of a biorthogonal functional group (i.e., an alkene tag) and its small size compared to e.g., pyrrolysine (Pyl) derivatives equipped with the same functional group. To date, Sac presents one of the smallest non-aromatic ncAAs which can be genetically encoded via the PylRS system. Moreover, it is a low-cost substrate with high pH-, temperature- and aqueous-stability (Kodera et al., 2002; Exner et al., 2017). Installed into peptides and proteins, Sac enables a variety of straightforward bioorthogonal protein conjugation reactions (Boutureira and Bernardes, 2015). Early GCE studies have revealed low *in vitro* solubility of the PylRS enzyme (Kavran et al., 2007). The low solubility is predominantly caused by the hydrophobic N-terminal domain which is essential for recognition, binding and charging of tRNA^Pyl^ *in vivo* (Herring et al., 2007). Subsequent works revealed improvements stemming from N-terminal mutations and/or exchange of the N-terminal domain of *Mb*PylRS by the more soluble counterpart of *Mm*PylRS, creating a chimeric aaRS enzyme (Bryson et al., 2017; Owens et al., 2017). These studies clearly indicate that there is potential for OTS improvement besides optimizing the enzymatic recognition and activation of substrates. Assuming that other components such as intracellular ncAA and orthogonal tRNA abundance are not limiting, poor recombinant aaRS solubility would translate into a lower fraction of active enzyme and concomitant lower OTS efficiency.

**FIGURE 1:**
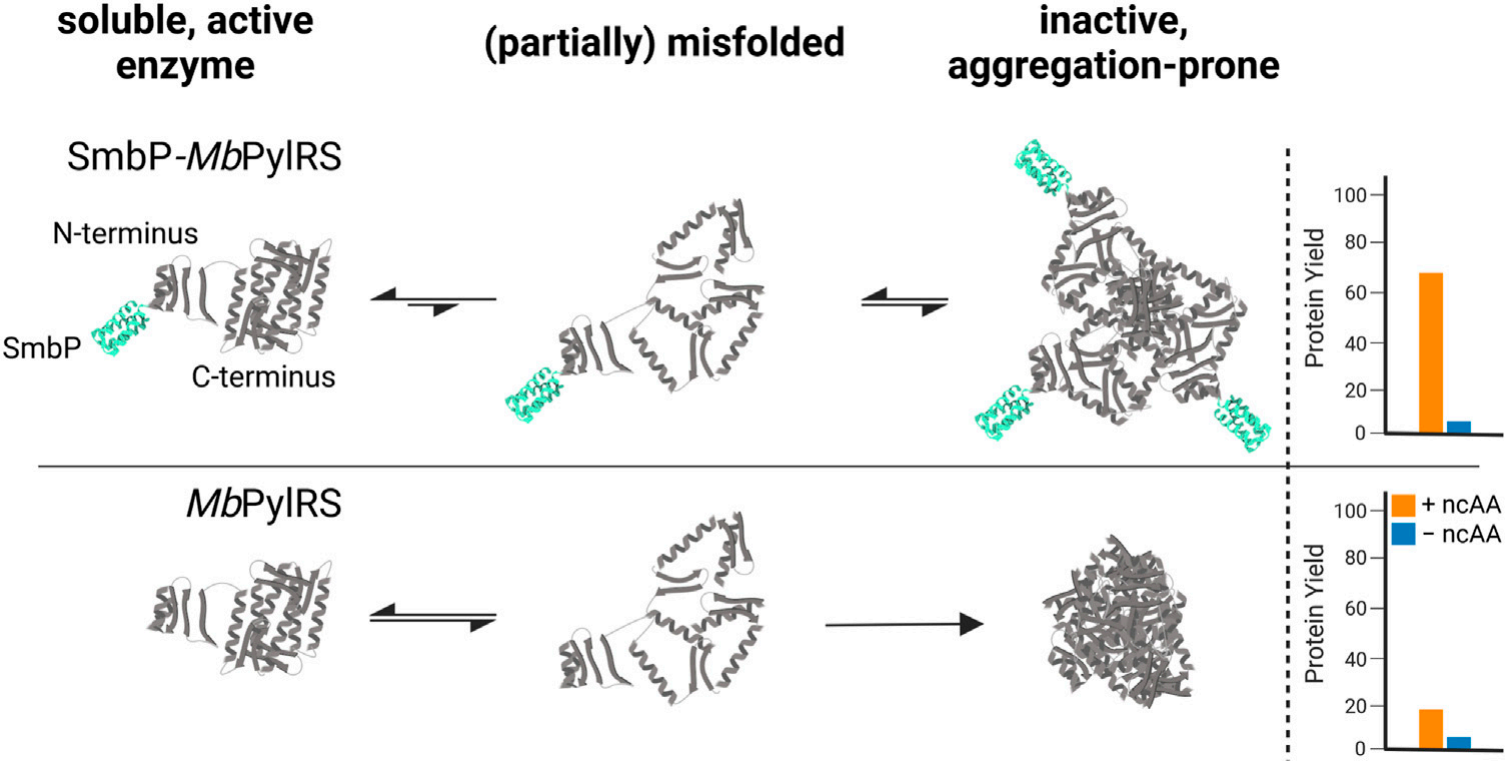
Proposed mechanism of how the SmbP-tag helps to provide more catalytically active *Mb*PylRS *in vivo*, increasing yield of ncAA-modified target protein.

**FIGURE 2:**
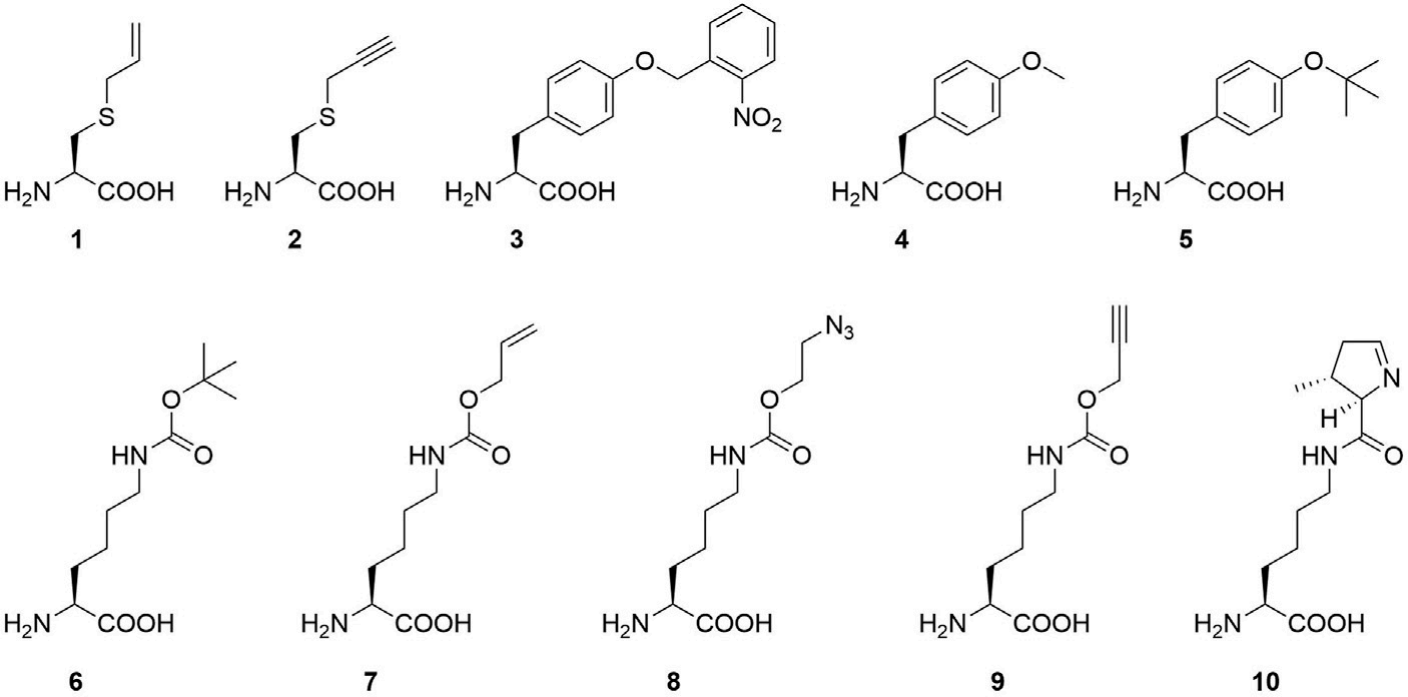
Overview of ncAAs used in this study (1-9) and the natural PylRS substrate (10): *S*-allyl-l-cysteine (Sac, 1), *S*-propargyl-l-cysteine (2), *o*-(2-nitrobenzyl)-l-tyrosine (ONBY, 3), (*O-*methyl-l-tyrosine (*O-*methyl-l-tyr, 4), *O-tert*-butyl-l-tyrosine (*O-tert*-butyl-l-tyr, 5), N^ε^-t*ert*-butoxycarbonyl-l-lysine (BocK, 6), N^ε^-allyloxycarbonyl-l-lysine (AllocK, 7), N^ε^-((2-azidoethoxy)carbonyl)-l-lysine (AzidoK, 8) and N^ε^-propargyloxycarbonyl-l-lysine (ProK, 9), Pyrrolysine (Pyl, 10).

Our strategy targets the N-terminus for alteration and our proposed working mechanism is shown in Figure 1. The active site and surrounding shell residues of the aaRS enzyme remain unaltered, facilitating transfer of successful findings to other enzymes. We selected a set of solubility-tags (Table 1), fused them to the N-terminus of a *Mb*PylRS enzyme variant previously engineered for the incorporation of *S*-allyl-l-cysteine (Sac, 1, Figure 2; Exner et al., 2017) and subsequently screened for improved efficiency. The generality of our findings was assessed using other engineered PylRS systems, increasing the production of site-specifically modified target proteins per amount of non-natural amino acid supplied.

**TABLE 1:** List of fusion tags used for solubility enhancement of *Mb*SacRS^a^ with brief descriptions, molecular weight (MW) and references.

Abbreviation	Full name and description	MW (kDa)	References
InfB (1–21)	First 21 nucleotides of the *E. coli* InfB gene encoding translation initiation factor IF-2. The mRNA secondary structure in the translation initiation region is weak, promoting ribosomal binding and high translation efficiencies	0.8	Hansted et al. (2011)
10xD	Ten aspartate residues: A polyanionic tag. The repulsive electrostatic interactions caused by the negative charge of the peptide tag are expected to enhance solubility and to facilitate correct protein folding by delaying protein aggregation	1.2	Rathnayaka et al. (2011)
10xR	Ten arginine residues: The improvement in protein solubility is attributed to the repulsive electrostatic interactions between similarly charged tags or protein stretches, which prevents aggregation and allows sufficient time for correct folding	1.5	Smith et al. (1984)
GB1	Immunoglobulin-binding domain B1 of protein G from group G *Streptococcus*: This domain possesses high thermal stability with a melting temperature (*T*_m_) of 87°C *in vitro* and completely reversible thermal denaturation, indicating excellent folding and solubility of the tag	6.2	Gronenborn et al. (1991)
Fh8	*Fasciola hepatica* antigen: 8 kDa calcium binding protein from the parasite *Fasciola hepatica*. Known to improve solubility of difficult-to-express recombinant target proteins upon fusion	8	Costa et al. (2013a), Costa et al. (2013b)
SmbP	Small metal-binding protein from *Nitrosomonas europaea*: A monomeric protein characterized by a series of 10 repeats of a seven amino acid motif with an unusually high number of histidine residues. Its unique sequence without homology to other proteins in current databases is considered to be a metal scavenging motif with an important role in cellular copper management	9.9	Vargas-Cortez et al. (2016)
SUMO	Small ubiquitin-related modifier: 100 AA residue protein which modulates protein structure and function by covalent modification of target proteins in eukaryotes. Well documented enhancer of recombinant protein expression and solubility	11.2	Malakhov et al. (2004)
Trx	*E. coli* thioredoxin: A small, ubiquitous protein with a dithiol-disulfide in an exposed active center. Thioredoxin facilitates reduction of various proteins through the reversible oxidation via cysteine thiol-disulfide exchange	11.8	Holmgren (1985)
NusA	*E. coli* N-utilization substance A: Predicted and found to enhance cytoplasmic solubility of target proteins in *E. coli* using a statistical solubility model	55	Davis et al. (1999)

a The MbSacRS fusion partner is 419 AA (47.5 kDa) in size.

## Materials and Methods

### Plasmid Vector Construction

All plasmids were assembled by Golden Gate cloning and confirmed by DNA sequencing. Plasmids harboring the OTS (aaRS/tRNA^Pyl^) were constructed by cloning the target aaRS gene into the pTECH vector (Bryson et al., 2017) (a gift from D. Söll and David R. Liu, Addgene plasmid #104073). Trx- and NusA-tag genes were amplified from chromosomal *E. coli* DNA. Fh8-and SmbP-tag DNA sequences were codon optimized and synthesized (GeneArt, Thermo Fisher Scientific). InfB (1-21)-, 10xD- and 10xR-tags were introduced upstream of the *Mb*SacRS gene via PCR amplification with the tag encoded in the forward primer overhang. Other tags were amplified from the research group’s internal plasmid database. Reporter plasmids were constructed in a previous work (Hauf et al., 2017).

### Analysis of sfGFP Expression by Intact Cell Fluorescence

For the small-scale expression of reporter constructs, *E. coli* BL21 (DE) cells were used. Electrocompetent cells were transformed with the orthogonal translation system and reporter plasmids. LB agar plates contained 1% glucose and corresponding antibiotics. Single colonies were used for inoculation of 2 ml LB (in 14 ml tubes) with 1% glucose and antibiotics and grown to saturation overnight. Assays were conducted in 96-well plate format. Cultures were added to each well at 1:100 dilution in ZYP-5052 auto induction medium to a final volume of 100 μL supplemented with antibiotics and ncAAs. Cells were grown in black μ-plates (Greiner Bio-One) covered with a gas permeable foil (Breathe-Easy®, Diversified Biotech) with orbital shaking for 24 h at 37°C. For endpoint measurements (Tecan M200), the foil was removed and fluorescence measured (fixed gain = 85). Excitation and emission wavelengths for fluorescence measurements were set to 481 and 511 nm, respectively. For OD_600_ measurements, 50 µL of ZYP-5052 medium was introduced into clear 96-well μ-plates and 50 µL culture added. Fluorescence values were normalized to the corresponding OD_600_ data. Relative fluorescence was normalized to the highest value. The data (incl. standard deviation) represents the mean of three biological replicates.

### Protein Expression

For expression of the SUMO-sfGFP variants, *E. coli* strain BL21 (DE) was used in 50 ml ZYP-5052 medium supplemented with 2 mM Sac and appropriate antibiotics. The expression medium was inoculated with a starter culture (1:100). Shake flasks were incubated for 20 h at 37°C, 200 rpm. Cells were harvested by centrifugation and stored at −80°C or directly used for protein purification.

### Protein Purification

Harvested cell pellets were resuspended (50 mM sodium phosphate, 300 mM NaCl, 20 mM imidazole, pH 8.0) and lysed by high-pressure homogenization (M-110L Microfluidizer, Microfluidics). After centrifugation (18,000 g, 60 min, 4°C), cleared lysates were loaded onto equilibrated Ni-NTA columns and purified *via* a peristaltic pump (Pharmacia Biotech, Stockholm, SE). After washing with 10 column volumes of resuspension buffer, elution buffer (50 mM sodium phosphate, 300 mM NaCl, 500 mM imidazole, pH 8.0) was applied to elute the his-tagged target proteins. The first 2 ml (covering the dead volume) were discarded. The eluate was collected in 2 ml fractions. After taking SDS samples, fractions were pooled and dialyzed in cellulose film tubings against 1 L buffer (50 mM sodium phosphate, 300 mM NaCl, pH 8.0) for at least 1.5 h with three buffer changes. Concentrations of purified reporter proteins were determined by measuring the sfGFP chromophore absorption at 488 nm.

### Analysis of Soluble and Insoluble Protein Fractions

*E. coli* strain BL21 (DE) was used in 2 ml ZYP-5052 medium supplemented with 2 mM Sac (to match target protein expression conditions) in 14 ml round bottom tubes (Falcon, Corning Life Sciences) and appropriate antibiotics. The expression medium was inoculated with a starter culture (1:100) and incubated for 20 h at 37°C, 200 rpm. 80 µL of culture were normalized to OD_600_ = 6 via dilution and absorption measurements with an Ultrospec 6300 pro spectrophotometer (Amersham Biosciences). Cells were harvested by centrifugation and lysed with B-PER® Bacterial Protein Extraction Reagent (Thermo Scientific) according to protocol, with addition of phenylmethanesulfonyl fluoride (1 mM final). Lysed cells were centrifuged (15,000 g, 5 min, RT) and the supernatant isolated (soluble protein fraction). The precipitate (insoluble protein fraction) was resolved (50 mM Tris-HCl, 7.5 M Urea, pH 8) with the same volume as the initial lysis buffer. Afterwards, soluble and insoluble protein fractions were combined with 5x SDS loading dye (80 mM Tris pH 6.8, 10% SDS, 12.5% glycerol, 4% (v/v) β-mercaptoethanol, 0.2% (w/v) bromophenol blue) and 5 µL samples were used for 15% acrylamide gels. PageRuler Prestained Protein Ladder or PageRuler Unstained Protein Ladder (both Thermo Scientific) were used. Electrophoresis was performed at 80 V for 60 min and afterwards at 120 V with SDS running buffer (190 mM glycine, 25 mM Tris, 3.5 mM SDS). Gels were stained with Coomassie solution overnight and destained with dH_2_O prior to documentation.

### ESI-MS

Intact protein mass measurements of purified SUMO-sfGFP variants were performed by electrospray LC-MS on a Waters H-class instrument with a Waters Acquity UPLC protein BEH C4 column (300 Å, 1.7 μm, 2.1 × 50 mm, gradient at a flow rate of 0.3 ml/min: A: 0.01% formic acid in H_2_O; B: 0.01% formic acid in MeCN. 5–95% B 0–6 min). Mass analysis was conducted with a Waters Xevo G2-XS QTof analyzer (positive mode, cone voltage = 40 kV). Raw data were analyzed employing the maximum entropy deconvolution algorithm and plotted with Origin.

## Results

### Choice and Comparison of Fusion Tags

To address the intrinsic solubility problem of the widely used PylRS scaffold, we focused on the *Methanosarcina barkeri* enzyme (*Mb*PylRS), whose solubility is even lower compared to *Mm*PylRS (Kavran et al., 2007). We intentionally chose an engineered enzyme and not the wild-type, as we assumed that if aaRS solubility/folding are major bottlenecks for *in vivo* function, the beneficial effects should be more pronounced for relatively inefficient enzyme variants. The chosen variant is a double mutant reported to yield 0.6 mg (per liter of bacterial culture) enhanced green fluorescent protein modified with Sac at a single site (Exner et al., 2017). Its two active site mutations C313W:W382S crucial for activation of the ncAA and two additional beneficial N-terminal mutations T13I:I36V (identified previously, cf. Supplementary Figure S5) were introduced into the codon-optimized *Mb*PylRS sequence (leading to the *Mb*SacRS variant). Mutation Y349F was also included by default, as it is known to generally enhance aminoacylation (Yanagisawa et al., 2008). This construct forms the reference OTS of this study.

We chose to test nine common protein fusion partners with diversity in size and physico-chemical properties (Table 1). We assumed that by keeping the sequence of the aaRS enzyme unaltered, the ncAA specificity and activation kinetics are maintained. To evaluate the efficiency of the OTS, we used a superfolder GFP (sfGFP)-based fluorescence readout. In this well-established methodology, the signal intensity of intact cells is directly correlated to the amount of produced protein. The reporter construct contains a stop codon replacing the R2 codon (sfGFP(R2 amber)) or three stop codons replacing the codons of R2, N39 and K101 (sfGFP(3x amber)), respectively. These reporters were co-expressed with the OTS using *E. coli* BL21 (DE3) as a widely used microbial production strain. With a RF1 positive strain (*prfA* ^*+*^), amber suppression competes with the endogenous translation termination machinery. This leads to the formation of truncation products when the activity of the OTS *in vivo* is too low to supply sufficient amounts of ncAA-charged orthogonal tRNA.

**FIGURE 3:**
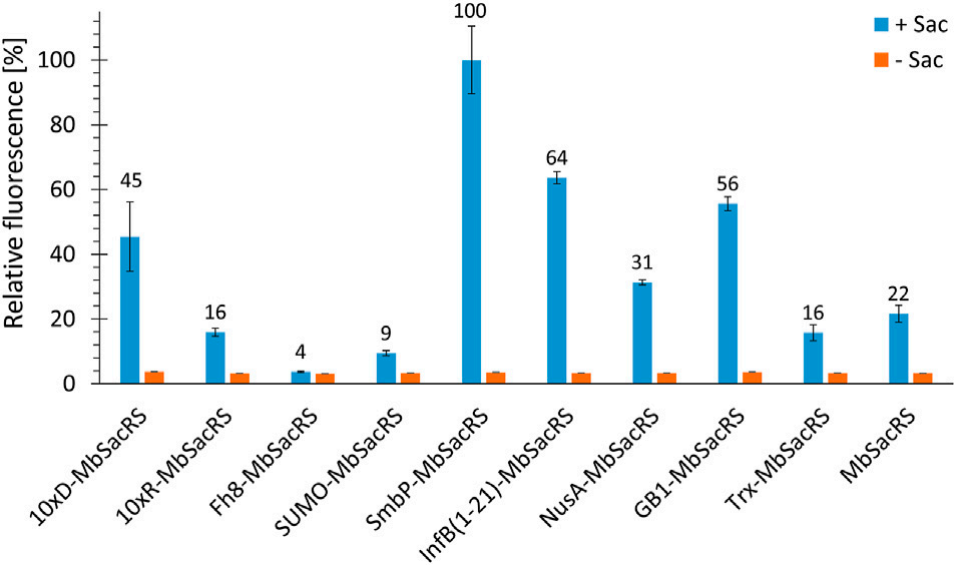
OTS efficiency comparison of 10 different *Mb*SacRS constructs measured via intact cell fluorescence of *E. coli* BL21 (DE3) expressing the sfGFP(R2 amber) construct. Reporter signal stems from stop codon suppression in presence (+Sac = 2 mM) or absence of ncAA supplementation (−Sac).

Comparing *Mb*SacRS as reference to nine fusion proteins with solubility tags shows that all constructs except for Fh8-*Mb*SacRS are functional *in vivo* (Figure 3). This is evident from increased fluorescence intensities in presence of ncAA supplementation. The best performing construct has an N-terminal SmbP-tag (9.9 kDa in size), followed by the InfB (1–21) and 10xD-tag.

**FIGURE 4:**
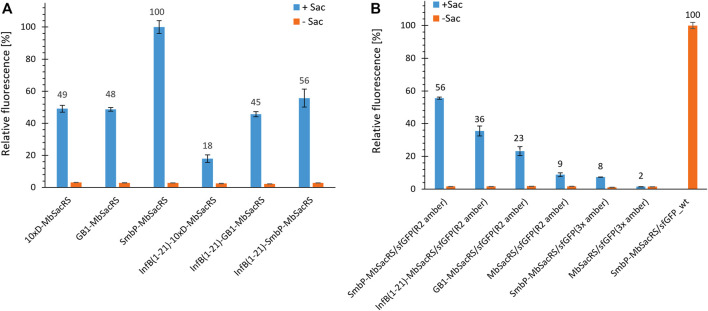
Comparison of OTS efficiency for *Mb*SacRS fusion constructs measured by the fluorescence of intact *E. coli* BL21 (DE3) cells. **(A)** Best-performing solubility tags were tested alone and in combination. **(B)** Comparison of fusion constructs to the untagged starting enzyme. Reporter constructs for the suppression of one or three stop codons were sfGFP(R2 amber) and sfGFP(3x amber), respectively. Wild-type sfGFP without an in-frame stop codon was included as benchmark. Endpoint measurements in presence (+Sac = 2 mM) or absence of ncAA supplementation (−Sac).

Given the increased protein production efficiency, we next aimed to check if even higher improvements could be achieved by using multiple tags. We chose to combine tags with a different mode of action, being InfB (1–21) on the one hand and SmbP, GB1 and 10xD on the other. Unfortunately, no synergistic effects could be observed (Figure 4A). After screening for the effect of different tags, we set out to characterize the performance of the three best constructs in detail. Wild-type sfGFP served as reference for maximum recombinant protein production. Based on fluorescence intensities, the overall efficiency for the suppression of one in-frame amber stop codon reaches around 56% (Figure 4B). This level of performance is very high especially for a PylRS system, far exceeding commonly reached levels for stop codon suppression at this position (Bryson et al., 2017; Exner et al., 2017). This encouraged us to evaluate if even more than one in-frame stop codon could now be efficiently suppressed. The best aaRS construct (SmbP-*Mb*SacRS) was co-expressed with a sfGFP gene containing three in-frame stop codons. Protein production reached the same level as the untagged starting enzyme (*Mb*SacRS) achieved for suppression of a single stop codon (Figure 4B). Co-expression of untagged *Mb*SacRS did not lead to a detectable suppression of three stop codons.

### Analytics of Sac Incorporation

**FIGURE 5:**
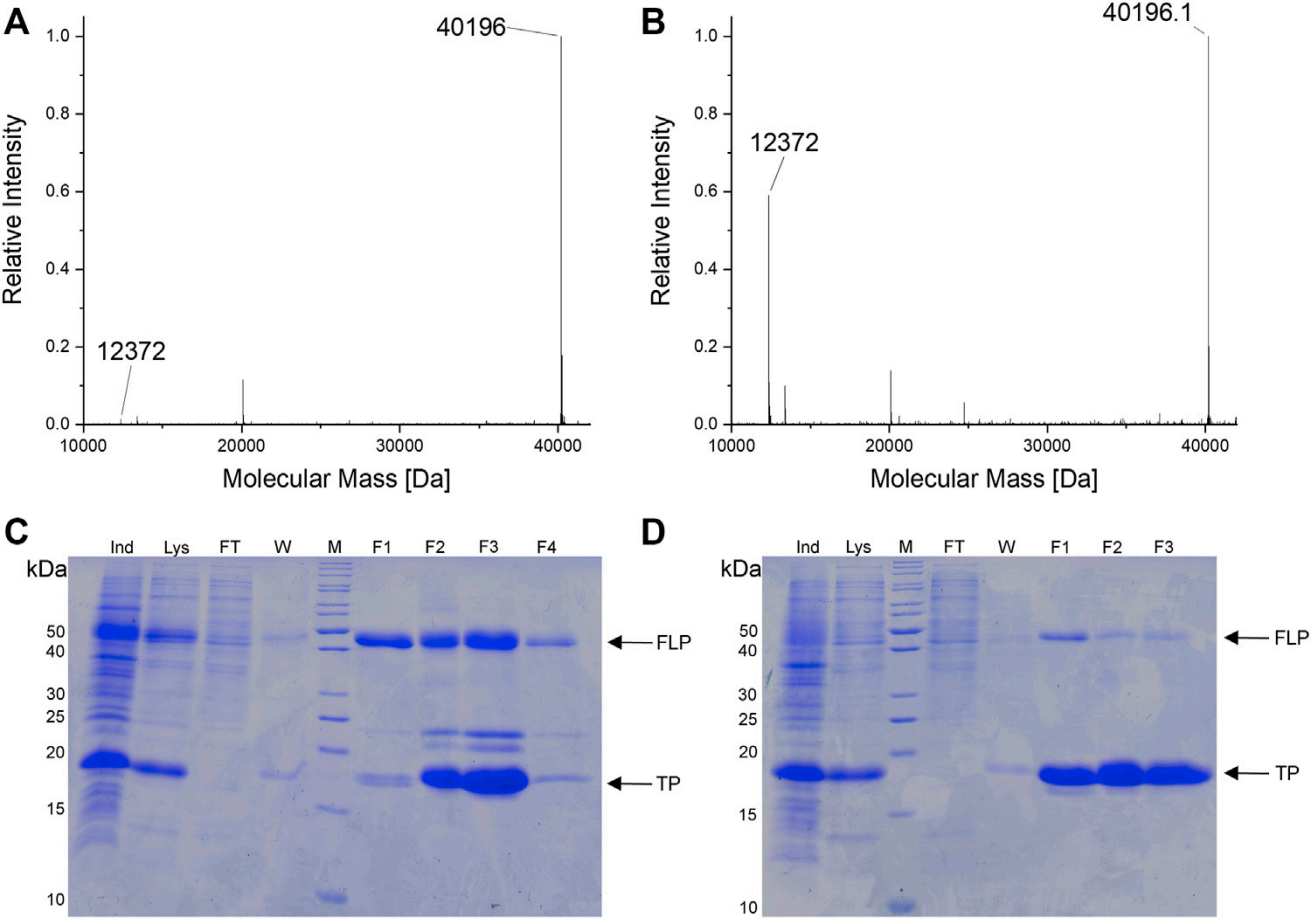
**(A,B)** Deconvoluted ESI-MS spectra of SUMO-sfGFP(R2Sac) produced in *E. coli* BL21 (DE3). **(A)** Reporter co-expression with SmbP-*Mb*SacRS. Expected protein mass of SUMO-sfGFP(R2Sac): 40,194.9 Da. Observed mass: 40,196 Da. **(B)** Co-expression with untagged *Mb*SacRS. Expected mass of SUMO sfGFP(R2Sac): 40,194.9 Da. Observed mass: 40,196.1 Da. Expected mass of SUMO truncation product: 12,372.8 Da. Observed mass: 12,372 Da (A and B). **(C,D)** SDS-PAGE analysis of purified SUMO-sfGFP(R2Sac) protein produced via co-expression of **(C)** SmbP-*Mb*SacRS and **(D)** unmodified *Mb*SacRS. Sample abbreviations: Whole cell extract of induced culture (Ind), soluble cellular lysate (Lys), liquid chromatography flow-through (FT), column wash of bound protein (W), eluate fractions (F1-3/4), protein ladder (M), full-length product (FLP), truncation product (TP).

To confirm the microtiter-scale intact cell fluorescence assays, the reporter constructs were expressed in bacterial cells in shake flasks and subsequently purified to determine target protein yields. This is the key metric for OTS performance and reflected by corresponding ratios between full-length target protein and truncation products. The reporter construct with an N-terminal His_6_-SUMO-tag allows simultaneous purification of both protein species. Cells carrying the improved SmbP-*Mb*SacRS OTS were likewise used to produce the positive control (wild-type sfGFP without an in-frame stop codon), to maintain a similar metabolic burden for *E. coli* cells. Addition of the ncAA to these cells had a negligible effect on the fluorescence signal (data not shown). The presence of Sac in the target protein was confirmed via electrospray ionization mass spectrometry (ESI-MS) (Figures 5A,B). Obtained purified protein yields for the different setups are in good agreement with data from the fluorescence assays (Table 2).

**TABLE 2:** Purified yields of ncAA-modified target protein as measure for OTS efficiency with 2 mM sac provided in the growth medium.

Reporter construct	aaRS construct	Protein yield (mg L^−1^)^a^
sfGFP wild-type	SmbP-*Mb*SacRS	40.2
sfGFP(R2 amber)	SmbP-*Mb*SacRS	15.2
sfGFP(R2 amber)	*Mb*SacRS	1.2
sfGFP(3x amber)	SmbP-*Mb*SacRS	0.8

a The amount of protein is given in mg per liter of bacterial culture after purification and dialysis.

The difference in reporter protein production is clearly visible by the naked eye (Supplementary Figure S4). Suppression of a single amber codon yielded purified protein amounts equivalent to about 40% of wild-type, highlighting the improved performance of the SmbP-tagged PylRS enzyme. Using ESI-MS, almost no truncation products were detected for purified samples obtained with this co-expression setup (Figure 5A). This also strongly emphasizes the higher efficiency of this PylRS construct. Increased amounts of full-length target protein are further evident from SDS-PAGE analysis when compared to the starting aaRS (Figures 5C,D). For the challenging construct with three in-frame amber stop codons, SDS-PAGE analysis indicates incorporation of three Sac moieties, but also reveals a predominant formation of truncation products (Supplementary Figure S2). This follows our expectations, as the amount of truncation product increases with the number of suppressed stop codons (Hauf et al., 2017). The relatively low amount of full-length, triple-modified target protein led us to switch to a C-terminally His-tagged version for selective purification. MS analysis confirmed the incorporation of Sac at three positions (Supplementary Figure S1).

**FIGURE 6:**
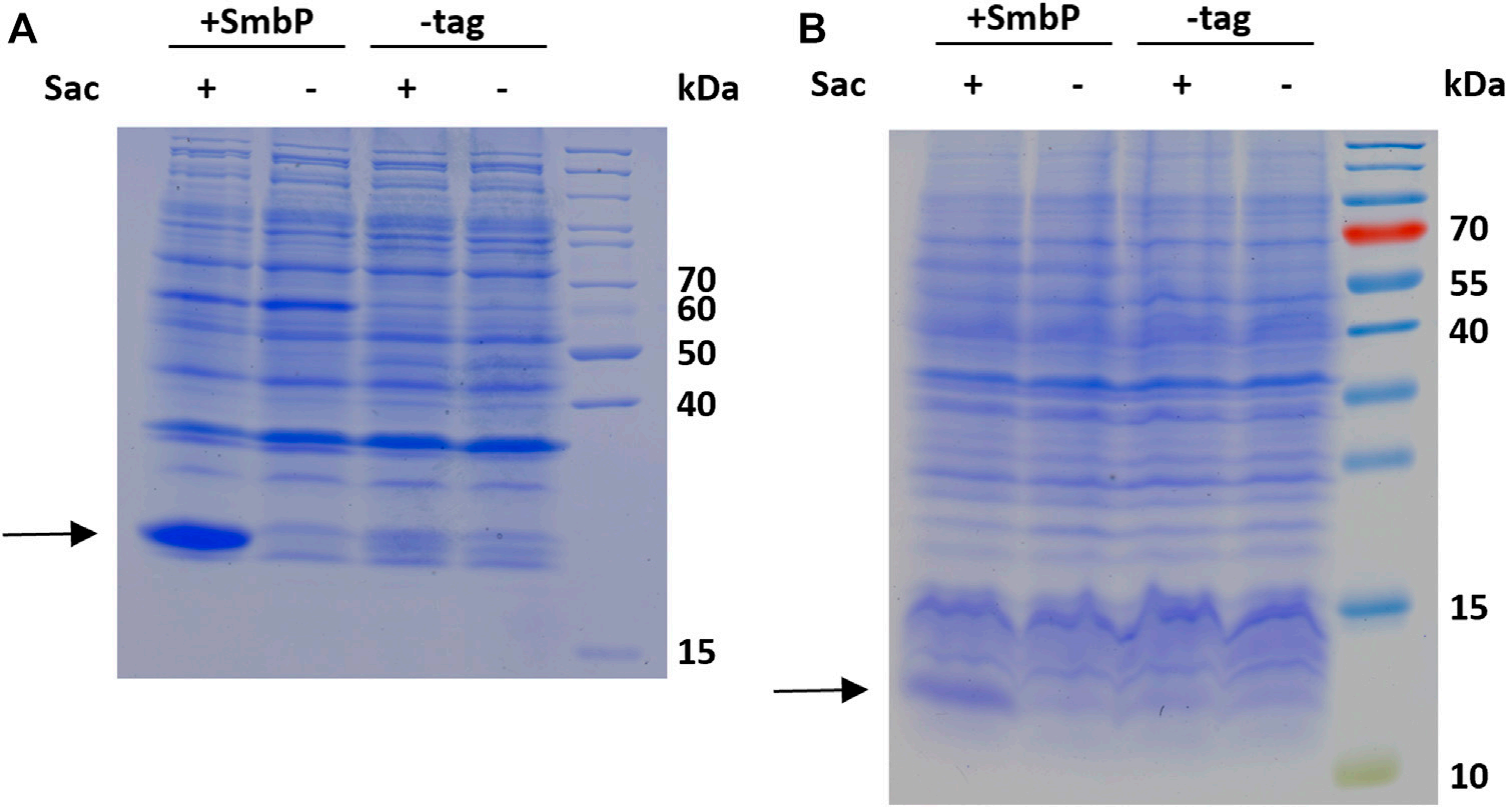
SDS-PAGE analysis of Sac incorporation into amilCP 26.7 kDa, **(A)** and PDZ 11.7 kDa, **(B)**. The gels show samples of the soluble whole cell lysates of induced cultures produced with 2 mM Sac supplementation (+) and without (−), respectively.

To evaluate the robustness and transferability of our findings, we decided to incorporate Sac into two other sequence contexts and protein structures. First construct is the blue chromoprotein from the coral *Acropora millepora* (amilCP, also a beta barrel in structure as sfGFP) where Sac was incorporated into the sequence context of an N-terminal tag (**6**-R11-1) evolved for highly efficient amber suppression (Figure 6A) (Pott et al., 2014). To evaluate Sac incorporation in a different context of overall protein structure, we employed the PDZ3 domain of postsynaptic density protein-95 (PSD-95) (Figure 6B).

### Analysis of PylRS Solubility

**FIGURE 7:**
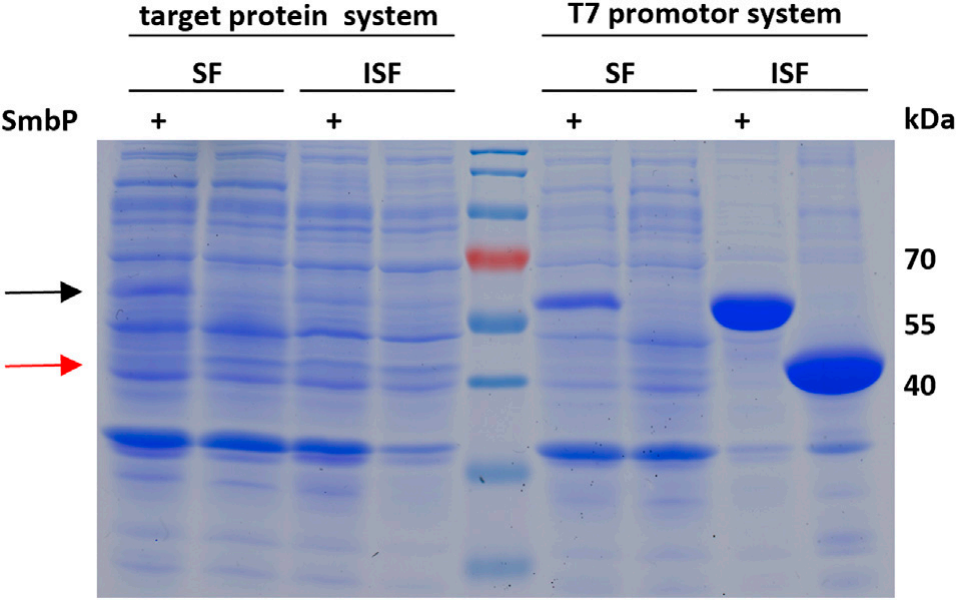
SDS-PAGE analysis of the soluble (SF) and insoluble (ISF) protein fractions of whole cell lysates. Left half: aaRS co-expression setup (driven by lpp promotor) as used for ncAA incorporation. Right half: aaRS overproduction setup driven by the strong T7 promoter. Calculated molecular weights: SmbP-*Mb*SacRS = 57.5 kDa (black arrow), *Mb*SacRS = 47.6 kDa (red arrow).

To confirm that the SmbP-tag increases the abundance of soluble enzyme in the cytoplasm of *E. coli*, we analyzed the soluble and insoluble cell fractions for SmbP-*Mb*SacRS and *Mb*SacRS expression via SDS-PAGE (Figure 7). For analytical reasons, we included an aaRS production setup with strong overexpression to facilitate detection. Accordingly, the aaRS gene was transferred into a pET plasmid vector with a strong T7 promotor. SmbP-*Mb*SacRS is clearly overproduced in soluble form, whereas untagged *Mb*SacRS is hardly detectible in the corresponding cellular fraction. In the same fashion, the aaRS co-expression setup was analyzed. For the lpp promotor driving aaRS production, clear overproduction of SmbP-*Mb*SacRS in the soluble cell extract fraction is visible; just a small band is detectable in the corresponding insoluble fraction.

### Transferability of Tag Effects to Other PylRS Variants

**FIGURE 8:**
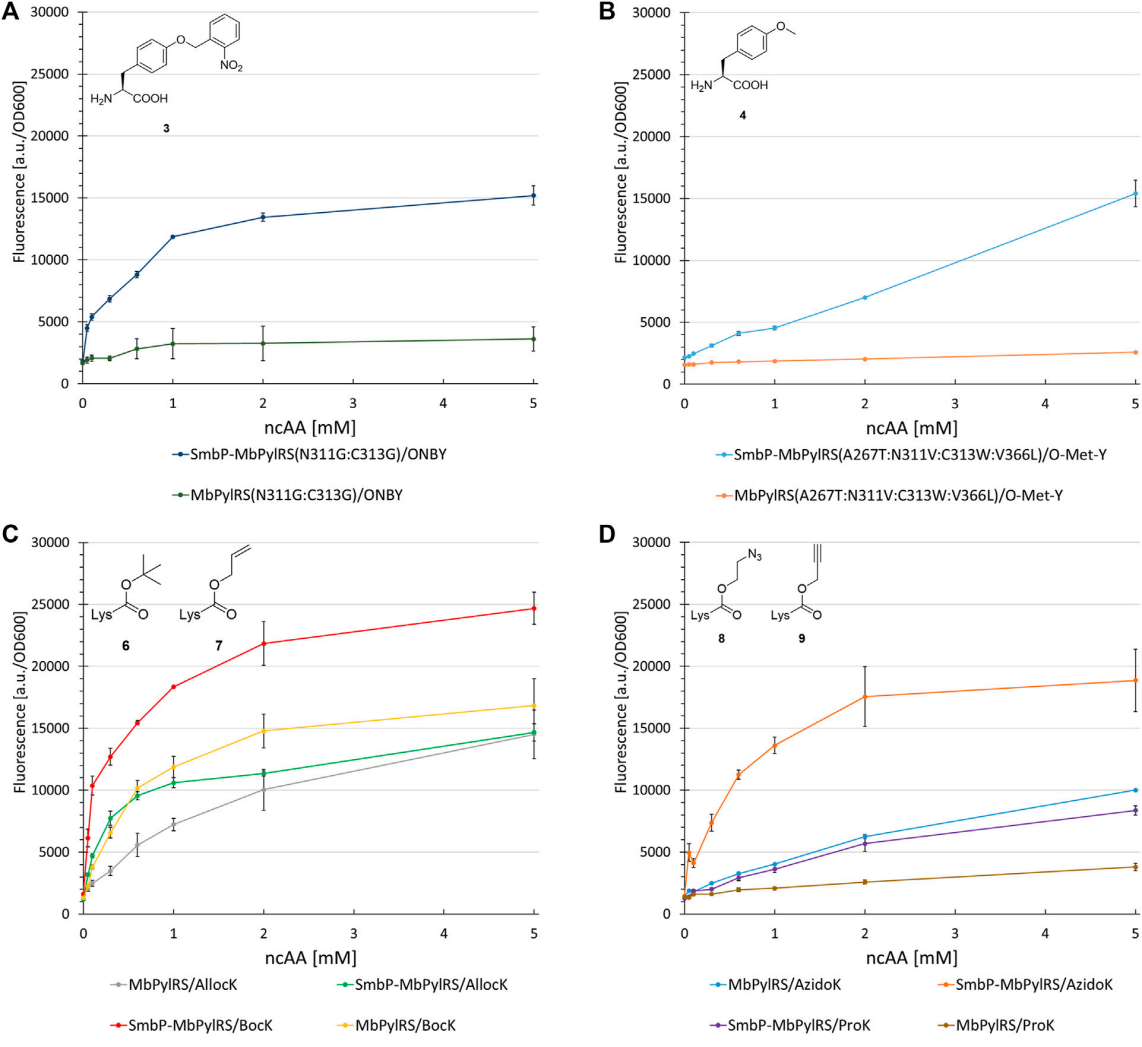
Concentration dependent unnatural protein production for different combinations of SmbP-*Mb*PylRS/*Mb*PylRS co-expression and ncAAs. Endpoint measurements for ncAA concentrations of 0.05, 0.1, 0.3, 0.6, 1, 2, and 5 mM.

To investigate the generality of our N-terminal tagging strategy, we equipped different *Mb*PylRS variants with the SmbP-tag. Paired with the corresponding engineered enzyme, we tested the following ncAA substrates in a concentration dependent manner to gain detailed information about the OTS performance *in vivo*. First candidate was a *Mb*PylRS variant engineered for incorporation of ONBY (Arbely et al., 2012). ONBY is a versatile photocaged tyrosine used for light-controlled protein activation. The aaRS enzyme has two glycines among a total of four catalytical domain mutations (L270F:L274M:N311G:C313G). It further allows genetic encoding of bulky, reversibly photoswitchable azobenzene amino acids (Luo et al., 2018). The SmbP-tag drastically improved protein production for ONBY (320%, Figure 8A). Mutations of aaRS active sites can result in an increased catalytic promiscuity (Wang et al., 2012). This is especially true for the hydrophobic amino acid binding pocket of PylRS, which is scarce in specific substrate interactions. Due to the enlarged binding pocket, we considered and confirmed the double glycine mutations to allow the accommodation of other ncAA substrates (Supplementary Figures S6, S7). We further investigated PylRS variants evolved for activation of a small substrate 4 (Figure 8B) and 2 (used as handle for the site specific azide-alkyne HUISGEN cycloaddition reaction, Supplementary Figure S8), respectively (Takimoto et al., 2011; Liu et al., 2018). Gratifyingly, increased protein yields were reached (200–490%) upon aaRS tagging.

Revealing the transferability of our approach to other engineered *Mb*PylRS variants encouraged us to test if even the wild-type enzyme could profit from our N-terminal tagging strategy. The ncAAs BocK (6), AllocK (7), AzidoK (8), and ProK (9) were screened in a concentration-dependent manner. Like 2, AzidoK and ProK are commonly used as handles for the site specific and bioorthogonal azide-alkyne HUISGEN cycloaddition reaction but possess longer side chains. All four ncAAs are established PylRS substrates with BocK more efficiently activated and charged to tRNA^Pyl^ compared to the other three. Protein production reached 170% (0.1 mM BocK) and 220% (0.6 mM AllocK) (Figure 8C), 245% (0.6 mM AzidoK) and 120% (2 mM ProK) (Figure 8D), highlighting that even the wild-type enzyme profits from the SmbP-tag.

### Impact of Active Site Mutations

Most natural enzymes are marginally stable at physiological temperatures. Recently, this has been specifically shown for *Mb*PylRS (Hu et al., 2020). Enzyme active site residues are inherently unfavorable for overall protein stability and mutations which drastically change the size and/or physico-chemical properties will likely aggravate this destabilization (Tokuriki et al., 2008b). To investigate our hypothesis that this holds true for the mutations enabling Sac incorporation (*via Mb*SacRS and its impactful C313W:W382S mutations), we created two control variants of the enzyme. The mutations for these constructs targeted the polar active site residues N311 and C313. These residues are most frequently chosen for PylRS engineering as they are responsible for the recognition of the native pyrrolysine substrate and have also proven to create a range of enzymes with new ncAA substrate recognition (Wan et al., 2014).

**FIGURE 9:**
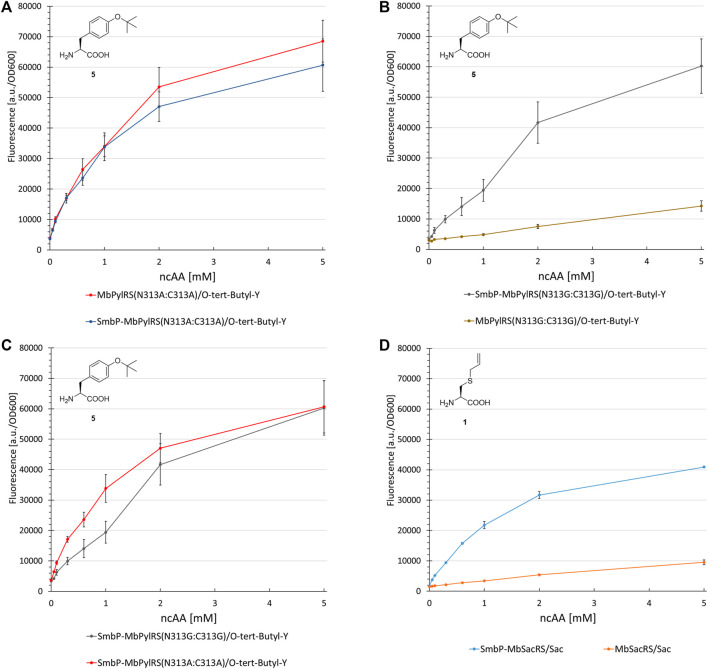
Concentration dependent unnatural protein production for different combinations of SmbP-*Mb*PylRS/*Mb*PylRS co-expression and ncAAs. Endpoint measurements for ncAA concentrations of 0.05, 0.1, 0.3, 0.6, 1, 2 and 5 mM.

The first control construct (where the SmbP-tag should have little to no effect) possesses the PylRS mutations N311A and C313A. The second construct (where mutations should severely destabilize the enzyme and therefore render the tag most effective) is the corresponding glycine double mutant, which was also used for ONBY incorporation (see above). Our assumptions were confirmed since no improvements could be detected for the SmbP-tagged double alanine mutant (Figure 9A and Supplementary Figure S9). In contrast, protein production with the double glycine construct increased markedly, by 450% (2 mM ncAA). This gain is comparable to the *Mb*SacRS improvement (540%, 1 mM Sac, cf. Figures 9B,D). Accepted by both enzyme variants, 5 was a suitable substrate for this comparison.

## Discussion

To test our initial hypothesis that the low solubility of engineered *Mb*PylRS can be remedied, a set of genetically encoded solubility tags derived from diverse organisms with diversity in size and physico-chemical properties was tested for improved protein production (i.e., overall OTS performance)*.* Due to the inherent complexity of the protein folding process *in vivo*, it is difficult to predict which amino acid position, small terminal tags or protein fusions will affect target protein solubility and activity *a priori* (Cheng and Patel, 2004; Esposito and Chatterjee, 2006; Zhou and Wagner, 2010). We assumed that by keeping the sequence of the aaRS enzyme unaltered, the ncAA specificity and activation function should be maintained. The InfB (1-21)-tag is the smallest fusion partner (0.8 kDa) tested herein, known in literature to increases expression levels rather than the solubility of proteins. Strikingly, even small modifications (e.g., His_6_-tags for aaRS purification) were previously shown to reduce the *in vivo* activity of engineered *Mb*PylRS variants (Lammers et al., 2014). In contrast, we found that most of our *Mb*PylRS fusion constructs were highly active (Figure 3). The N-terminal SmbP-tag even boosts the performance of wild-type (120–245%) and especially engineered *Mb*PylRS systems (200–540%), therefore delivering more protein for the amount of ncAA supplied (Figure 8). Combining more than one tag did not lead to additional improvements, indicating that the activity increasing effects of the tags are non-additive (Figure 4A). Our collected data on single and combined tags do not reveal a clear pattern to rationalize the observed effects. Improvements in ribosomal ncAA incorporation could arise from enhanced aaRS expression levels, folding, solubility and combinations thereof. Three of the initially chosen tags reduced the efficiency compared to unmodified *Mb*SacRS, highlighting that the aaRS scaffold is sensitive to certain terminal modifications. Albeit the multi-faceted nature of protein folding and solubility, previous studies have shown benefits of fusion partners which are highly robust towards thermal and chemical unfolding (like GB1 Gronenborn et al., 1991). Moreover, it should be noted that both on nucleotide and amino acid level, the N-terminal sequence plays an important role for the initiation and efficiency of protein synthesis, which could promote changes in aaRS expression levels (Verma et al., 2019).

The robustness of ncAA incorporation was additionally assessed with the Sac incorporation into two additional sequence contexts and protein structures. First, the blue chromoprotein from the coral *Acropora millepora* where we incorporated Sac into the sequence context of an N-terminal tag (**6-**R11-1) evolved for highly efficient amber suppression (Pott et al., 2014). With flanking glycine residues, this small region would be expected to be unstructured and solvent-exposed (Figure 6A). To evaluate Sac incorporation in a different context of protein structure, we employed the PDZ3 domain of PSD-95 (Figure 6B). PDZ domains are of special interest as they represent highly abundant protein-protein interaction modules. These structural domains are found in the proteins of a variety of signal transduction complexes in multiple organisms (bacteria, yeast, plants and viruses). Taking the mouse genome as an example, they can be found 928 times (Doyle et al., 1996; Lee and Zheng, 2010). Sac was successfully incorporated at position F325, a location previously used for ncAA installation and part of the peptide ligand binding pocket (Baumann et al., 2019). The results of Sac incorporation into amilCP and PDZ are consistent with the previous sfGFP results (Figure 5). There is clearly more target protein production in the system with the SmbP-tagged aaRS, in the case of the chromoprotein amilCP even visible in the harvested bacterial cells by the naked eye (Supplementary Figure S3).

To further evaluate our hypothesis that the improved performance stems from more soluble *Mb*PylRS, we performed a solubility analysis of cells which express the OTSs (Figure 7). When expressed with a strong T7 promotor, SDS-PAGE showed that most of the engineered aaRS with and without tag resides in the insoluble fraction. It is thus likely inactive *in vivo*, for instance present in bacterial inclusion bodies. The corresponding soluble fractions, however, displayed distinct differences in aaRS abundance. SmbP-*Mb*SacRS is clearly overproduced in soluble form. In contrast, *Mb*SacRS co-expression generates only a small detectible band in both fractions, indicating a relatively low functional aaRS production level. Based on the solubility results, it seems plausible that the SmbP-tag protects PylRS from pathways towards irreversible protein aggregation *in vivo*. Therefore, the tagged *Mb*PylRS does not become removed from the folding/misfolding equilibrium. Comparing the findings of the two aaRS promoter systems, it is noteworthy to mention that the total abundance of soluble SmbP-*Mb*SacRS appears comparable. This indicates that despite large differences in promoter strength, similar levels of soluble SmbP-*Mb*SacRS are produced within the cell.

Assessing the transferability of our approach, several *Mb*PylRS variants including the wild-type were fused to the SmbP-Tag (Figure 8). The performance of ONBY (2) incorporation via genetic code expansion was drastically improved, strikingly even though two second-shell mutations (L270F:L274M) were omitted. Not in direct contact to the ncAA substrate, we had chosen to focus just on the glycine double mutant. It is conceivable that the two side chain truncations are key for opening up the ncAA binding pocket towards accommodation of the bulky photocaged moiety. The impact of the N-terminal tag on aaRS efficiency and the obtained protein production (even at low supplied ncAA concentrations) was astonishing. The activity of SmbP-*Mb*PylRS(N311G:C313G) towards several ncAAs emphasizes the role of reducing the size of side chain moieties within the PylRS pocket. Similar observations were made upon rationally engineering the enzyme’s substrate tolerance via a double alanine mutation (Supplementary Figures S6, S7) (Wang et al., 2012).

Further investigation of PylRS variants evolved for activation of the small substrate 3 (Figure 8B), and 2 highlighted the detrimental effects of active site mutations. The SmbP-tag drastically increased the performance of these OTSs. Even the wild-type *Mb*PylRS profits from the tag, reaching up to 245% protein production. The efficiency observed for the tagged *Mb*PylRS with BocK as ncAA substrate supplied at very low concentrations is noteworthy. Almost half of the maximum protein production is already reached at 0.1 mM BocK (Figure 8C). This is one order of magnitude below the commonly used ncAA concentration and far from quantities used in the majority of studies, where up to 10 mM ncAA are required to reach satisfactory protein production yields (Liu et al., 2018; Willis and Chin, 2018; Hu et al., 2020). Comparing the genetic encoding of BocK and Sac, it is remarkable that the engineered SmbP-*Mb*SacRS system can achieve even higher target protein yields. Substrate titration revealed that the very low efficiency of the starting enzyme can be boosted to even surpass the wild-type enzyme performance for an excellent, established substrate (Figures 8C, 9D).

Lastly, the impact of active-site mutations in regard to OTS performance was elucidated (Figure 9). For this purpose, two aaRS variants were constructed containing two mutations which should either stabilize (N311A:C313A) or destabilize (N311G:C313G) the enzyme and therefore alter the OTS performance accordingly. It is known that changing polar/charged residues in the active site to alanine often enhances the stability of enzymes, but in most cases also leads to a decrease in catalytic activity (Beadle and Shoichet, 2002). In contrast, the introduction of glycine disrupts the secondary structure elements in most soluble proteins, due to its high backbone flexibility (Imai and Mitaku, 2005). Taken the *Mm*PylRS crystal structure as homology model and the transferability of active site mutations between *Mm*PylRS and *Mb*PylRS as basis, both mutation sites (N311 and C313) are part of the same β-sheet. They come in close contact with the ncAA substrate (<5 Å in PDB ID 3QTC; note that *Mb*PylRS could not be crystallized thus far). Placing two glycines into this defined secondary structure element should perturb the folding and stability of the enzyme given the conformational flexibility of the smallest amino acid residue and the different backbone torsion angle preferences (Imai and Mitaku, 2005). Removal of the N311 and C313 side chains should profoundly destabilize the local secondary and subsequently overall aaRS structure. Substrate 5 was used as model substrate in these experiments. This ncAA has been shown to work well as a structural reporter in NMR spectroscopy, but due to low protein yields of the PylRS system initially reported, only the use of a *Mj*TyrRS-based system in bacterial cells proved applicable (Chen et al., 2015). The protein yield reported for a similar reporter construct paired with the *M. mazei* double alanine mutant was 2 mg/L (Wang et al., 2012). On the basis of our reporter, protein yields exceed 15 mg/L (note that this is estimated for a supply of 1 mM ncAA in contrast to 5 mM in the original publication).

Our comprehensive data for a set of ncAA and aaRS enzyme pairs strongly supports our working hypothesis that intracellular folding and solubility of engineered PylRS enzymes easily becomes a bottleneck for orthogonal translation - besides the kinetics of ncAA binding, activation and charging to the cognate tRNA. Our findings show that the expression level and solubility of these enzymes are important parameters that should not be neglected when optimizing cells for the production of ncAA-modified unnatural proteins. In the light of previous reports and the reaction pathway towards charged tRNA, it was remarkable to see that even the attachment of large fusion partners did not abolish the *in vivo* function of the orthogonal pair, with the N-terminal aaRS domain primarily involved in tRNA recognition and binding.

## Conclusion

In this study, we rationally approached the low intrinsic protein solubility of PylRS enzymes and its impact on the production of ncAA-modified unnatural proteins. Altogether, we could demonstrate that the classic method of obtaining more soluble and active enzymes via fusion partners can be transferred to OTS engineering. Notably, this strategy to enhance aaRS performance (up to 540%) *in vivo* does not involve changes in the catalytic domain or active site. Our findings suggest that engineered enzymes with drastic active site changes particularly benefit from rescue by solubility tags. The tag appears to remediate the destabilizing effects of active site mutations initially introduced to allow genetic encoding of new ncAAs. This explains why the observed improvements are higher for engineered aaRS variants than for the wild-type enzyme. The improvements of the latter case highlight that the archaeal enzyme can be tailored for functional expression in recombinant hosts. Concomitantly, the fidelity of the ncAA incorporation system is preserved, as we do not detect increased background suppression. The most potent tag was shown to enhance the soluble expression of the enzyme, which is most likely the cause of the increased efficiency observed. We thus envision the transfer of this approach to more PylRS-based systems used *in vivo* or *in vitro*, where it could substantially boost the efficiency of unnatural protein production. Additionally, a generally increased aaRS activity and robustness could enable the engineering of enzymes able to activate new classes of ncAA substrates. It is reasonable to assume that many aaRS variants with suitable residues in the active site (generated by mutations) remain hidden to us due to insufficient aaRS stability/activity for detection by screening systems. Our approach could help to find these hidden variants. Additionally, the increased amount of obtained soluble *Mb*PylRS could finally facilitate the crystal structure elucidation of this enzyme.

## Data Availability

The original contributions presented in the study are included in the article/Supplementary Material, further inquiries can be directed to the corresponding author.
