# Supplementary material for: Efficient Unnatural Protein Production by Pyrrolysyl-tRNA Synthetase With Genetically Fused Solubility Tags
Source: Front Bioeng Biotechnol. 2021 Dec 23;9:807438. doi: 10.3389/fbioe.2021.807438 (PMC8905625; doi:10.3389/fbioe.2021.807438)
Supplement: Supplementary file 1 [file DataSheet1.pdf]

Supplementary Material

Efficient unnatural protein production by pyrrolysyl-tRNA synthetase with genetically fused solubility tags

Nikolaj G. Koch[1,2], Tobias Baumann[1], Nediljko Budisa[1,3*]

[1]Biokatalyse, Institut für Chemie, Technische Universität Berlin, Berlin, Germany

[2]Bioanalytik, Institut für Biotechnologie, Technische Universität Berlin, Berlin, Germany

[3]Chemical Synthetic Biology, Department of Chemistry, University of Manitoba, Winnipeg, Manitoba, Canada

Table of contents

1. Supplementary Results 3

2. DNA sequences used in this study 8

3. References 11

1. Supplementary Results

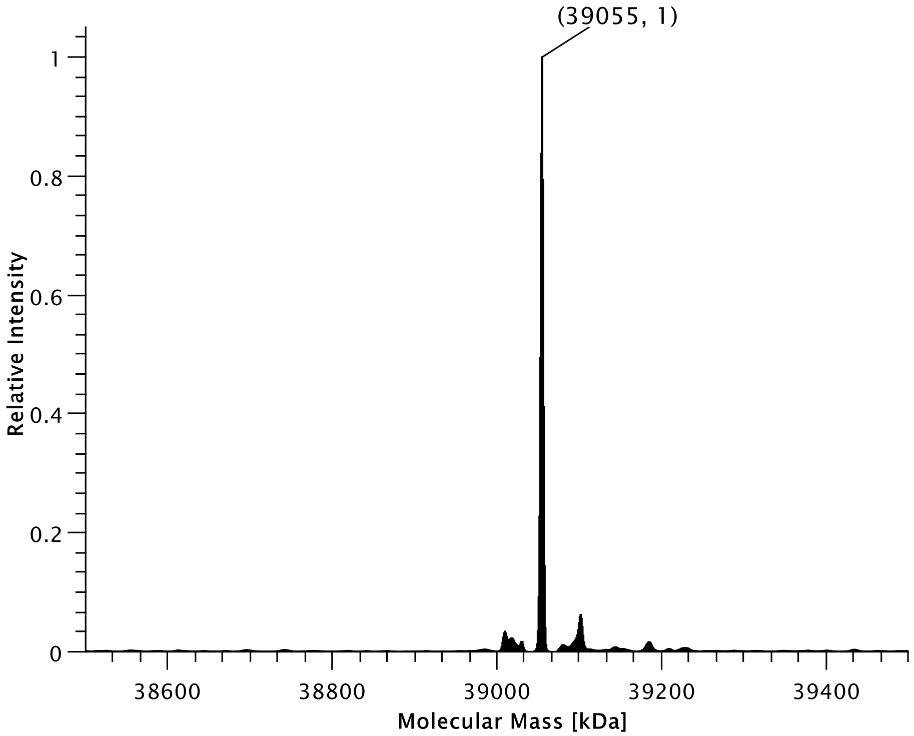

Supplementary Figure S1: Deconvoluted ESI-MS spectra of SUMO-sfGFP(R2Sac:N39Sac:K101Sac)-His_6 produced in *E. coli* BL21(DE3) with co-expression of SmbP-*Mb*SacRS. Expected protein mass: 39055.2 Da. Observed mass: 39055 Da.

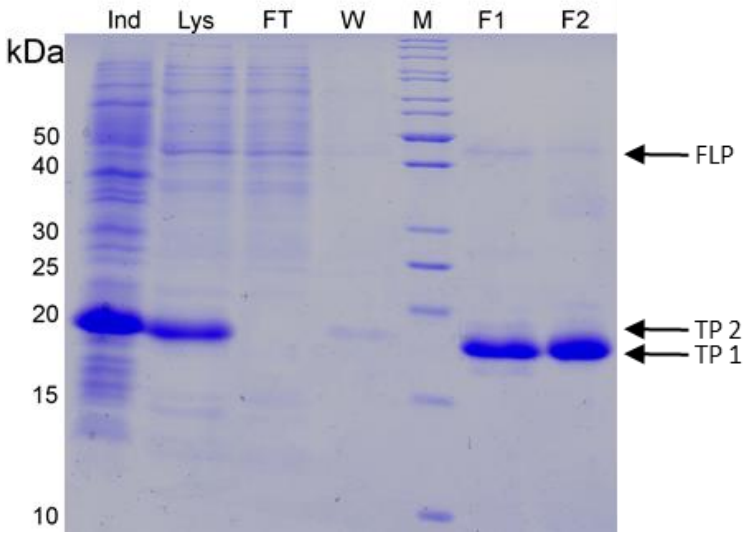

Supplementary Figure S2: SDS-PAGE analysis of purified His_6-SUMO-sfGFP(R2Sac:N39Sac:K101Sac) reporter expressed in *E. coli* BL21(DE3) with SmbP-*Mb*SacRS co-expression. Sample abbreviations: Whole cell extract of induced culture (Ind), soluble cellular lysate (Lys), liquid chromatography flow through (FT), column wash of bound protein (W), different fractions of eluate (F1-2), protein ladder (M), full length product (FLP), truncation product 1 (TP1), truncation product 2 (TP2).

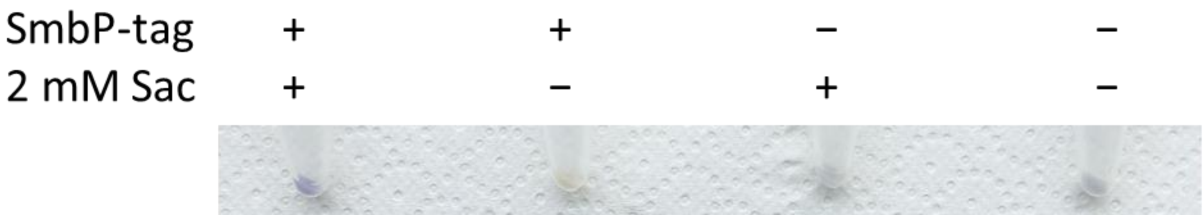

Supplementary Figure S3: OD_{600}-normalized, pelleted *E. coli* BL21(DE3) cells from amilCP target protein production with site-specific ncAA installation. Co-expression of the SmbP-tagged aaRS leads to visibly higher production of the blue chromoprotein.

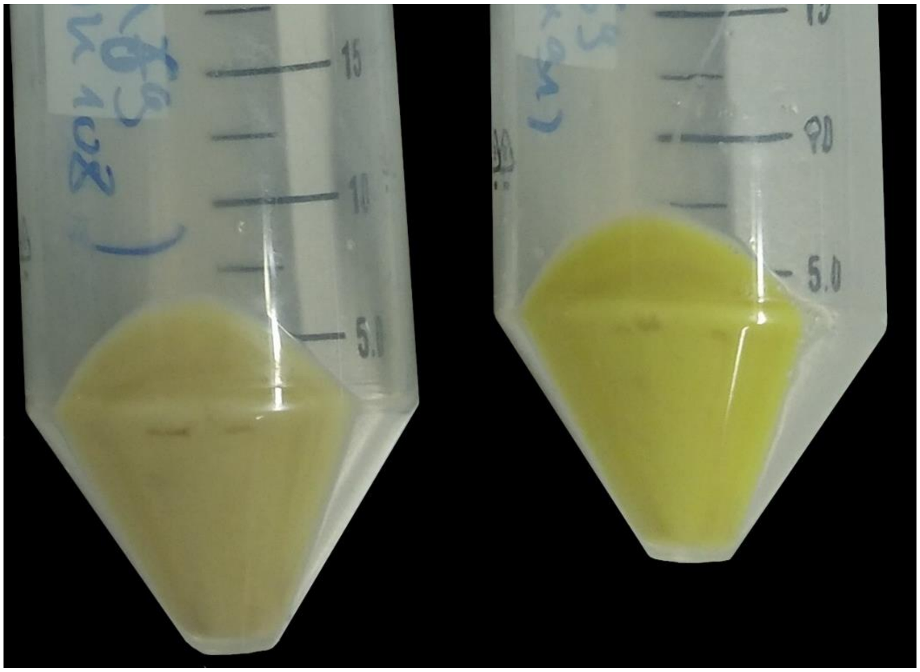

Supplementary Figure S4: Harvested *E. coli* BL21(DE3) cells after shake flask cultivation for production of Sac-modified sfGFP reporter protein. Untagged (left) vs. tagged (right) *Mb*SacRS OTS. Production of ncAA-modified target protein was conducted as described in the main text. Image background removal requested during review.

Previously published N-terminal PylRS mutations

In an earlier study, seven N-terminal mutations were shown to increase the ribosomal N^{ε}-crotonyl-lysine incorporation efficiency of an OTS based on an engineered *Mb*PylRS enzyme.(Owens et al., 2017) It was claimed that these mutations could generally increase ncAA incorporation efficiencies for PylRS-based systems, as they don't alter the aaRS substrate specificity towards ncAAs. Herein, four of these mutations were tested. Transferred to the OTS for ribosomal Sac incorporation, mutations T13I and I36V resulted in a modest enhancement of reporter production

in comparison to *Mb*SacRS lacking these mutations (**Figure S5**). Based on these results, the two mutations were incorporated by default in all *Mb*SacRS constructs. All tested PylRS variants carry the Y384F mutation by default (see main text).

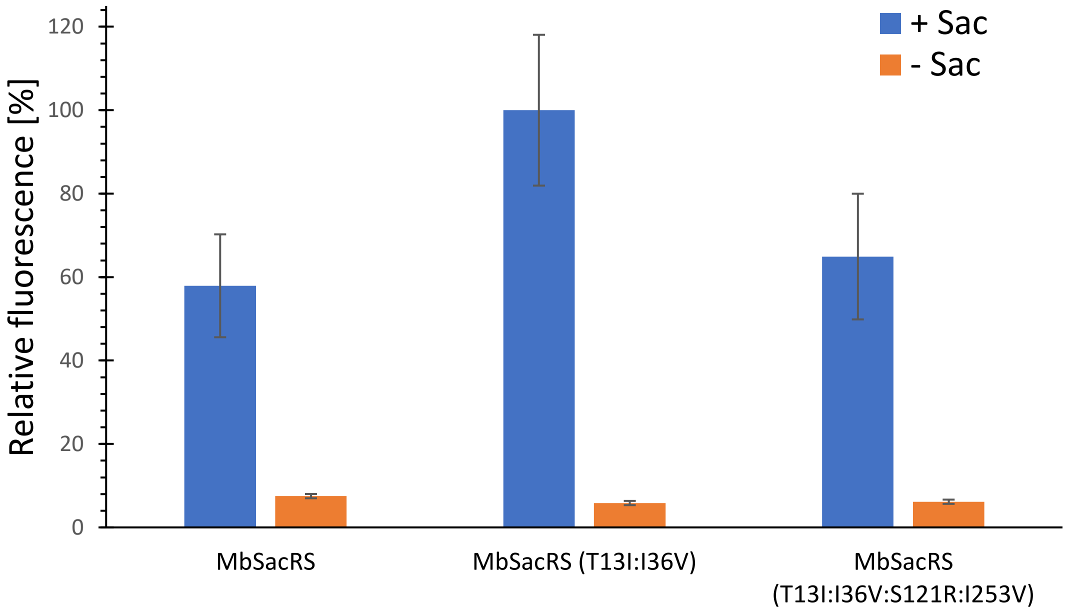

Supplementary Figure S5: OTS efficiency comparison of three different *Mb*SacRS constructs. Ribosomal incorporation of Sac (+ Sac = 2 mM) and controls without ncAA (- Sac) supplementation. Intact cell fluorescence of *E. coli* BL21(DE3), endpoint measurements after 24 h of incubation. Relative fluorescence is normalized to the highest value. The data (incl. standard deviation) represent the mean of three biological replicates.

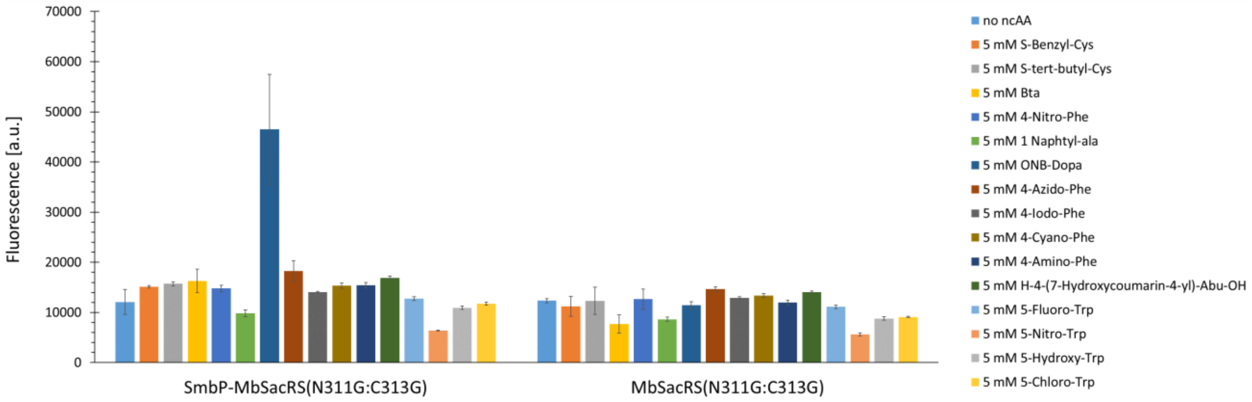

Supplementary Figure S6: OTS efficiency comparison of *Mb*PylRS(N311G:C313G) co-expression with and without SmbP-tag. Ribosomal incorporation of 15 different ncAAs. Intact cell fluorescence of *E. coli* BL21(DE3) cells expressing the sfGFP(R2 amber) reporter, endpoint measurements after 16 h of incubation for the presence or absence of ncAA supplementation. Relative fluorescence is normalized to the highest value. The data (incl. standard deviation) represent the mean of three biological replicates.

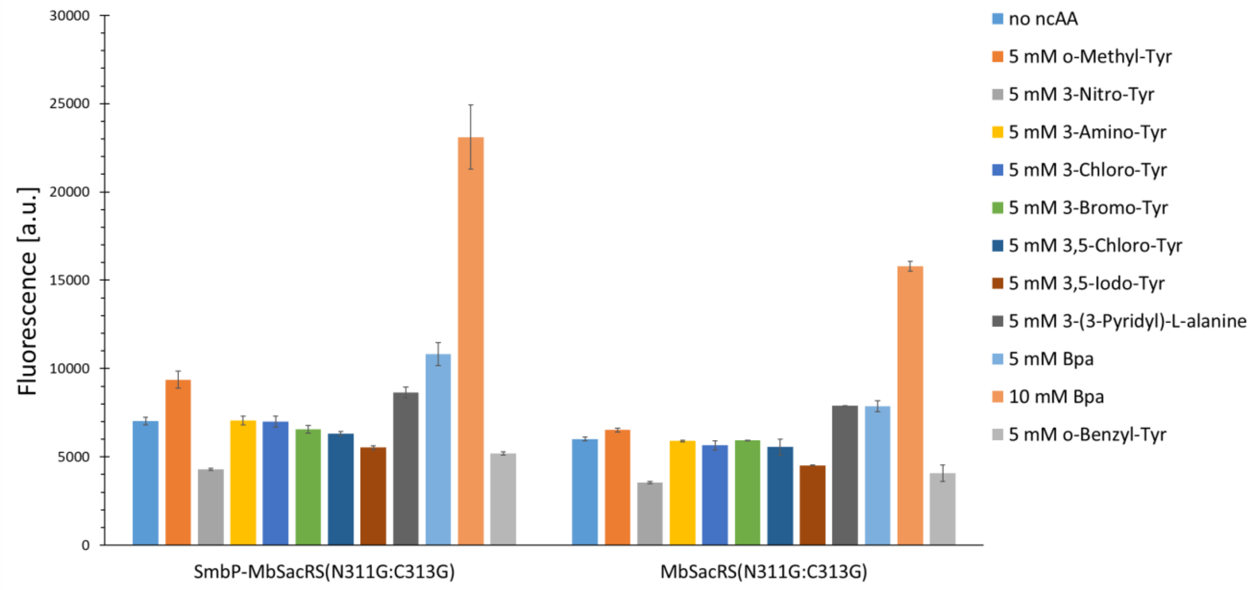

Supplementary Figure S7: OTS efficiency comparison of *Mb*PylRS(N311G:C313G) co-expression with and without SmbP-tag. Ribosomal incorporation of 11 different ncAAs. Measured fluorescence intensity of intact *E. coli* BL21(DE3) cells expressing the sfGFP(R2 amber) reporter, endpoint measurements after 24 h of incubation for the presence or absence of ncAA supplementation. Relative fluorescence is normalized to the highest value. The data (incl. standard deviation) represent the mean of three biological replicates.

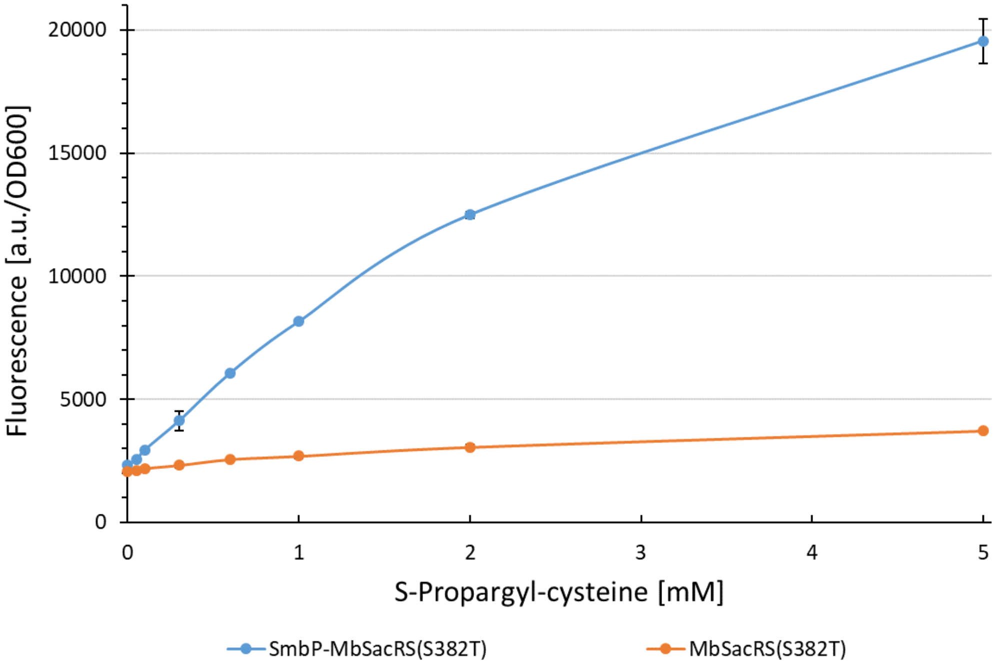

Supplementary Figure S8: Concentration dependent protein production for *Mb*PylRS with and without SmbP-tag. Measured fluorescence intensity of intact *E. coli* BL21(DE3) cells expressing the sfGFP(R2 amber) reporter, endpoint measurements after 24 h. Endpoint measurements with different ncAA concentrations (0.05, 0.1, 0.3, 0.6, 1, 2, and 5 mM) of *S*-Propargyl-L-cysteine.

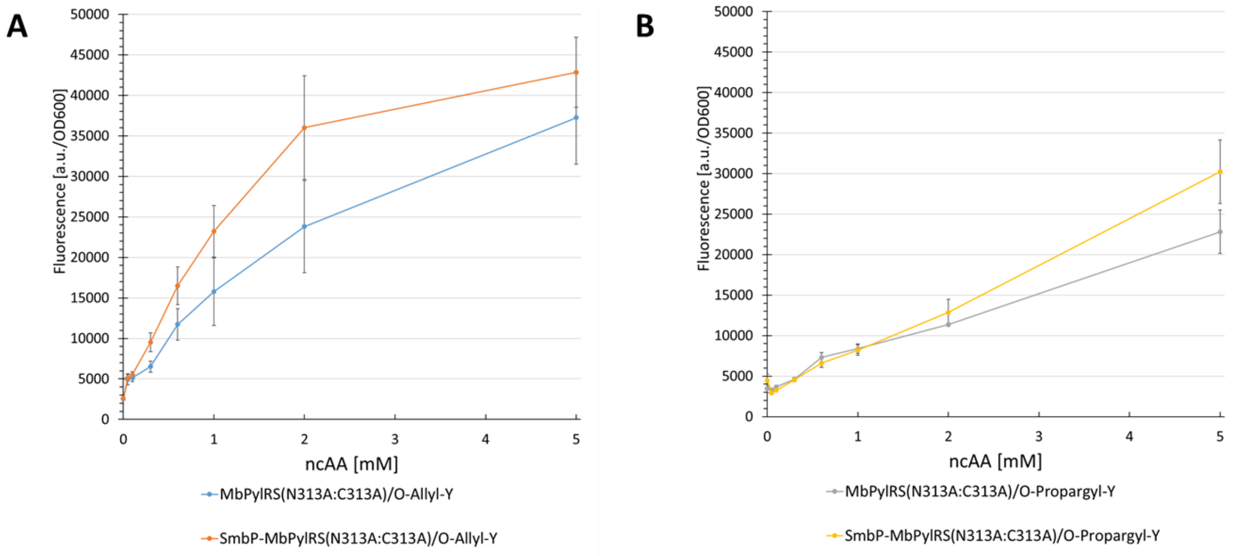

Supplementary Figure S9: Concentration dependent protein production for different MbPylRS/ncAA combinations. Measured fluorescence intensity of intact *E. coli* BL21(DE3) cells expressing the sfGFP(R2 amber) reporter, endpoint measurements after 24 h. Endpoint measurements with different ncAA concentrations (0.05, 0.1, 0.3, 0.6, 1, 2, and 5 mM). The ncAAs are: **A**) O-Allyl-Y = O-Allyl-L-tyrosine **B**) O-Propargyl-Y = O-Propargyl-L-tyrosine.

2. DNA sequences used in this study

The following section contains DNA sequences used in this study. For clarity, important regions of combined constructs are highlighted by color.

1) Starting aaRS variant *Mb*PylRS(T13I:I36V:C313W:Y349F:W382S), referred to as *Mb*SacRS in this study. The two mutations in the C-terminal aaRS domain C313W:W382S correlate with the earlier published *Methanosarcina mazei* based SacRS (*Mm*SacRS) mutations.(Exner et al., 2017)

ATGGACAAAAAACCGCTGGACGTTCTGATTAGCGCAATTGGTCTGTGGATGAGCCGTACCGGCACCCTGCATAAAATCAAACAT
CATGAAGTTAGCCGCAGCAAAGTCTATATTGAAATGGCATGTGGTGATCATCTGGTGGTGAATAATAGCCGTAGCTGTCGTACC
GCACGTGCATTTCGTCATCACAAATATCGTAAAACCTGTAAACGTTGCCGTGTTAGCGACGAAGATATTAACAATTTTCTGACCC
GTAGCACCGAAAGCAAAAATTCAGTTAAAGTTCGTGTTGTGAGCGCTCCGAAAGTTAAAAAAGCAATGCCGAAAAGCGTTAGTC
GTGCACCGAAACCTCTGGAAAATAGCGTTAGCGCAAAAGCAAGCACCAATACCAGCCGTAGCGTTCCGAGTCCGGCAAAAAGC
ACCCCGAATAGCAGCGTTCCGGCAAGCGCACCGGCACCGAGCCTGACCCGTTCACAGCTGGATCGTGTTGAAGCACTGCTGA
GCCCTGAAGATAAAATCAGCCTGAATATGGCAAAACCGTTTCGTGAACTGGAACCGGAACTGGTTACCCGTCGTAAAAATGATT
TTCAGCGTCTGTATACCAACGATCGCGAAGATTATCTGGGTAAACTGGAACGTGATATTACCAAATTTTTCGTGGATCGCGGTTT
TCTGGAAATCAAAAGCCCGATTCTGATTCCGGCAGAATATGTTGAACGTATGGGCATTAATAACGATACCGAACTGAGCAAACA
AATCTTCCGCGTTGATAAAAATCTGTGTCTGCGTCCGATGCTGGCACCGACCCTGTATAACTATCTGCGCAAACTGGATCGTATT
CTGCCTGGTCCGATTAAAATCTTTGAAGTTGGTCCGTGCTATCGCAAAGAAAGTGATGGTAAAGAACACCTGGAAGAGTTTACG
ATGGTTAACTTTTGGCAGATGGGTAGCGGTTGTACCCGTGAAAATCTGGAAGCACTGATTAAAGAGTTTCTGGACTATCTGGAA
ATTGACTTTGAAATTGTTGGCGATAGCTGCATGGTTTTTGGTGATACCCTGGATATTATGCATGGTGATCTGGAACTGAGTAGCG
CAGTTGTTGGTCCGGTTAGCCTGGATCGCGAATGGGGTATTGATAAACCGTCTATTGGTGCAGGTTTTGGTCTGGAACGTCTGC
TGAAAGTTATGCACGGCTTTAAAAACATTAAACGTGCAAGCCGTTCCGAGAGCTATTACAATGGTATTAGCACCAACCTGTAA

Containing the N-terminal ATG start codon, all tags were N-terminally fused to the *Mb*SacRS sequence. Tags 5-10 contain a GGSH-linker between solubility-tag and *Mb*SacRS.

GGSH-linker:

GGCGGCTCTCAT

2) InfB(1-21)-tag

ATGACAGATGTAACGATTAAA

3) 10xD-tag

ATGGATGATGACGACGATGACGATGATGATGAC

4) 10xR-tag

ATGAGACGTCGTAGACGTCGCCGTCGTCGTCGT

5) GB1-tag

ATGCAATACAAACTCATTCTCAATGGCAAGACGCTCAAGGGTGAAACCACGACCGAAGCGGTGAATGCGGCCACCGCGGAGAA
AGTGTTCAAACAGTATGCGAACGACAATGGCGTCAATGGCGAATGGACCTATGACGATGCGACCAAAACCTTCACCGTAACCG
AA

6) Fh8-tag

ATGCCGAGCGTTCAAGAAGTTGAAAAACTGCTGCATGTTCTGGATCGTAATGGTGATGGTAAAGTTAGCGCAGAAGAACTGAAA
GCATTTGCCGATGATAGCAAATGTCCGCTGGATAGCAATAAAATCAAGGCCTTTATCAAAGAGCACGATAAAAACAAAGATGGC
AAGCTGGATCTGAAAGAACTGGTTAGCATTCTGAGCAGC

7) SmbP-tag

ATGAGCGGTCATACCGCACATGTTGATGAAGCAGTTAAACATGCCGAAGAAGCAGTTGCACACGGTAAAGAAGGCCATACCGA
TCAGCTGCTGGAACATGCAAAAGAAAGTCTGACCCATGCCAAAGCAGCCAGCGAAGCCGGTGGTAATACCCATGTTGGTCATG
GTATTAAACATCTGGAAGATGCCATCAAACATGGTGAAGAGGGTCATGTTGGTGTTGCGACCAAACACGCACAAGAAGCAATTG
AACATCTGCGTGCAAGCGAACATAAAAGCCAT

8) Trx-tag

ATGAGCGATAAAATTATTCACCTGACTGACGACAGTTTTGACACGGATGTACTCAAAGCGGACGGGGCGATCCTCGTCGATTTC
TGGGCAGAGTGGTGCGGTCCGTGCAAAATGATCGCCCCGATTCTGGATGAAATCGCTGACGAATATCAGGGCAAACTGACCGT
TGCAAAACTGAACATCGATCAAAACCCTGGCACTGCGCCGAAATATGGCATCCGTGGTATCCCGACTCTGCTGCTGTTCAAAAA
CGGTGAAGTGGCGGCAACCAAAGTGGGTGCACTGTCTAAAGGTCAGTTGAAAGAGTTCCTCGACGCTAACCTGGCG

9) SUMO-tag

ATGGGTTCTGACTCCGAAGTCAATCAAGAAGCTAAGCCAGAGGTCAAGCCAGAAGTCAAGCCTGAGACTCACATCAATTTAAAG
GTGTCCGATGGATCTTCAGAGATCTTCTTCAAGATCAAAAAGACCACTCCTTTAAGAAGGCTGATGGAAGCGTTCGCTAAAAGA
CAGGGTAAGGAAATGGACTCCTTAAGATTCTTGTACGACGGTATTAGAATCCAAGCTGATCAGACCCCTGAAGATTTGGACATG
GAGGATAACGATATTATTGAGGCTCACCGCGAACAGATT

10) NusA-tag

ATGAACAAAGAAATTTTGGCTGTAGTTGAAGCCGTATCCAATGAAAAGGCGCTACCTCGCGAGAAGATTTTCGAAGCATTGGAA
AGCGCGCTGGCGACAGCAACAAAGAAAAAATATGAACAAGAGATCGACGTCCGCGTACAGATCGATCGCAAAAGCGGTGATTT
TGACACTTTCCGTCGCTGGTTAGTTGTTGATGAAGTCACCCAGCCGACCAAGGAAATCACCCTTGAAGCCGCACGTTATGAAGA
TGAAAGCCTGAACCTGGGCGATTACGTTGAAGATCAGATTGAGTCTGTTACCTTTGACCGTATCACTACCCAGACGGCAAAACA
GGTTATCGTGCAGAAAGTGCGTGAAGCCGAACGTGCGATGGTGGTTGATCAGTTCCGTGAACACGAAGGTGAAATCATCACCG
GCGTGGTGAAAAAAGTAAACCGCGACAACATCTCTCTGGATCTGGGCAACAACGCTGAAGCCGTGATCCTGCGCGAAGATATG
CTGCCGCGTGAAAACTTCCGCCCTGGCGACCGCGTTCGTGGCGTGCTCTATTCCGTTCGCCCGGAAGCGCGTGGCGCGCAAC
TGTTCGTCACTCGTTCCAAGCCGGAAATGCTGATCGAACTGTTCCGTATTGAAGTGCCAGAAATCGGCGAAGAAGTGATTGAAA
TTAAAGCAGCGGCTCGCGATCCGGGTTCTCGTGCGAAAATCGCGGTGAAAACCAACGATAAACGTATCGATCCGGTAGGTGCT
TGCGTAGGTATGCGTGGCGCGCGTGTTCAGGCGGTGTCTACTGAACTGGGTGGCGAGCGTATCGATATCGTCCTGTGGGATG
ATAACCCGGCGCAGTTCGTGATTAACGCAATGGCACCGGCAGACGTTGCTTCTATCGTGGTGGATGAAGATAAACACACCATG
GATATCGCCGTTGAAGCCGGTAACCTGGCGCAGGCGATTGGCCGTAACGGTCAGAACGTGCGTCTGGCTTCGCAGCTGAGCG
GTTGGGAACTCAACGTGATGACCGTTGACGACCTGCAGGCTAAGCATCAGGCGGAAGCGCACGCAGCGATCGACACCTTCAC

CAAATATCTCGACATCGACGAAGACTTCGCGACTGTTCTGGTAGAAGAAGGCTTCTCGACGCTGGAAGAATTGGCCTATGTGCC
GATGAAAGAGCTGTTGGAAATCGAAGGCCTTGATGAGCCGACCGTTGAAGCACTGCGCGAGCGTGCTAAAAATGCACTGGCCA
CCATTGCACAGGCCCAGGAAGAAAGCCTCGGTGATAACAAACCGGCTGACGATCTGCTGAACCTTGAAGGGGTAGATCGTGAT
TTGGCATTCAAACTGGCCGCCCGTGGCGTTTGTACGCTGGAAGATCTCGCCGAACAGGGCATTGATGATCTGGCTGATATCGA
AGGGTTGACCGACGAAAAAGCCGGAGCACTGATTATGGCTGCCCGTAATATTTGCTGGTTCGGTGACGAAGCG

11) His$_6$-SUMO-sfGFP(R2 amber)-strep reporter construct

ATGGGCAGCAGCCATCATCATCATCATCACGGTTCTGACTCCGAAGTCAATCAAGAAGCTAAGCCAGAGGTCAAGCCAGAAGT
CAAGCCTGAGACTCACATCAATTTAAAGGTGTCCGATGGATCTTCAGAGATCTTCTTCAAGATCAAAAAGACCACTCCTCTGCGT
CGTCTGATGGAAGCGTTCGCTAAAAGACAGGGTAAGGAAATGGACTCCTTAAGATTCTTGTACGACGGTATTAGAATCCAAGCT
GATCAGACCCCTGAAGATTTGGACATGGAGGATAACGATATTATTGAGGCTCATCGCGAACAGATTGGTGGCATGTAGAAAGG
CGAAGAGCTGTTCACTGGTGTCGTCCCTATTCTGGTGGAACTGGATGGTGATGTCAACGGTCATAAGTTTTCCGTGCGTGGCG
AGGGTGAAGGTGACGCAACTAATGGTAAACTGACGCTGAAGTTCATCTGTACTACTGGTAAACTGCCGGTACCTTGGCCGACT
CTGGTAACGACGCTGACTTATGGTGTTCAGTGCTTTGCTCGTTATCCGGACCATATGAAGCAGCATGACTTCTTCAAGTCCGCC
ATGCCGGAAGGCTATGTGCAGGAACGCACGATTTCCTTTAAGGATGACGGCACGTACAAAACGCGTGCGGAAGTGAAATTTGA
AGGCGATACCCTGGTAAACCGCATTGAGCTGAAAGGCATTGACTTTAAAGAAGACGGCAATATCCTGGGCCATAAGCTGGAATA
CAATTTTAACAGCCACAATGTTTACATCACCGCCGATAAACAAAAAAATGGCATTAAAGCGAATTTTAAAATTCGCCACAACGTG
GAGGATGGCAGCGTGCAGCTGGCTGATCACTACCAGCAAAACACTCCAATCGGTGATGGTCCTGTTCTGCTGCCAGACAATCA
CTATCTGAGCACGCAAAGCGTTCTGTCTAAAGATCCGAACGAGAAACGCGATCATATGGTTCTGCTGGAGTTCGTAACCGCAGC
GGGCATCACGCATGGTATGGATGAACTGTACAAAAGCGCTTGGAGCCACCCGCAGTTCGAAAAATAA

12) His$_6$-SUMO-sfGFP(3x amber)-strep reporter construct

ATGGGCAGCAGCCATCATCATCATCATCACGGTTCTGACTCCGAAGTCAATCAAGAAGCTAAGCCAGAGGTCAAGCCAGAAGT
CAAGCCTGAGACTCACATCAATTTAAAGGTGTCCGATGGATCTTCAGAGATCTTCTTCAAGATCAAAAAGACCACTCCTCTGCGT
CGTCTGATGGAAGCGTTCGCTAAAAGACAGGGTAAGGAAATGGACTCCTTAAGATTCTTGTACGACGGTATTAGAATCCAAGCT
GATCAGACCCCTGAAGATTTGGACATGGAGGATAACGATATTATTGAGGCTCATCGCGAACAGATTGGTGGCATGTAGAAAGG
CGAAGAGCTGTTCACTGGTGTCGTCCCTATTCTGGTGGAACTGGATGGTGATGTCAACGGTCATAAGTTTTCCGTGCGTGGCG
AGGGTGAAGGTGACGCAACTTAGGGTAAACTGACGCTGAAGTTCATCTGTACTACTGGTAAACTGCCGGTACCTTGGCCGACT
CTGGTAACGACGCTGACTTATGGTGTTCAGTGCTTTGCTCGTTATCCGGACCATATGAAGCAGCATGACTTCTTCAAGTCCGCC
ATGCCGGAAGGCTATGTGCAGGAACGCACGATTTCCTTTTAGGATGACGGCACGTACAAAACGCGTGCGGAAGTGAAATTTGA
AGGCGATACCCTGGTAAACCGCATTGAGCTGAAAGGCATTGACTTTAAAGAAGACGGCAATATCCTGGGCCATAAGCTGGAATA
CAATTTTAACAGCCACAATGTTTACATCACCGCCGATAAACAAAAAAATGGCATTAAAGCGAATTTTAAAATTCGCCACAACGTG
GAGGATGGCAGCGTGCAGCTGGCTGATCACTACCAGCAAAACACTCCAATCGGTGATGGTCCTGTTCTGCTGCCAGACAATCA
CTATCTGAGCACGCAAAGCGTTCTGTCTAAAGATCCGAACGAGAAACGCGATCATATGGTTCTGCTGGAGTTCGTAACCGCAGC
GGGCATCACGCATGGTATGGATGAACTGTACAAAAGCGCTTGGAGCCACCCGCAGTTCGAAAAATAA

13) **6**-R11-1-amilCP-his

ATGGGTCATCATTAGAAGAATGGTGGTGCTAGTGTGATCGCTAAACAAATGACCTACAAGGTTTATATGTCAGGCACGGTCAAT
GGACACTACTTTGAGGTCGAAGGCGATGGAAAAGGTAAGCCCTACGAGGGGGAGCAGACGGTAAAGCTCACTGTCACCAAGG
GCGGACCTCTGCCATTTGCTTGGGATATTTTATCACCACAGTGTCAGTACGGAAGCATACCATTCACCAAGTACCCTGAAGACA
TCCCTGACTATGTAAAGCAGTCATTCCCGGAGGGCTATACATGGGAGAGGATCATGAACTTTGAAGATGGTGCAGTGTGTACTG
TCAGCAATGATTCCAGCATCCAAGGCAACTGTTTCATCTACCATGTCAAGTTCTCTGGTTTGAACTTTCCTCCCAATGGACCTGT
CATGCAGAAGAAGACACAGGGCTGGGAACCCAACACTGAGCGTCTCTTTGCACGAGATGGAATGCTGCTAGGAAACAACTTTA
TGGCTCTGAAGTTAGAAGGAGGCGGTCACTATTTGTGTGAATTTAAAACTACTTACAAGGCAAAGAAGCCTGTGAAGATGCCAG

GGTATCACTATGTTGACCGCAAACTGGATGTAACCAATCACAACAAGGATTACACTTCGGTTGAGCAGTGTGAAATTTCCATTGC
ACGCAAACCTGTGGTCGCCGGCAGCCATCATCATCATCATCACTAA

6-R11-1 contains an amber codon and flanking glycine residues.(Pott et al., 2014) Parts of the amilCP coding sequence were derived from the iGEM Registry of Standard Biological Parts (http://parts.igem.org/Part:BBa_K592009).

14) PDZ(F325amber)-his

ATGGGTGGTGAAGAAGATATTCCTCGCGAACCGCGTCGTATTGTTATTCATCGTGGTAGCACCGGTCTGGGTTAGAATATCATT
GGTGGTGAAGATGGCGAAGGCATTTTTATTAGCTTTATTCTGGCAGGCGGTCCGGCAGATCTGAGCGGTGAACTGCGTAAAGG
TGATCAGATTCTGAGCGTTAATGGTGTTGATCTGCGTAATGCAAGCCATGAACAGGCAGCAATTGCACTGAAAAATGCAGGTCA
GACCGTTACCATTATCGCACAGTATAAACCGGAAGAATATAGCCGTTTTGAAGCAGGCAGCCATCATCATCATCATCACTAA

Details of this construct have been published before.(Baumann et al., 2019)

3. References

Baumann, T., Hauf, M., Schildhauer, F., Eberl, K. B., Durkin, P. M., Deniz, E., et al. (2019). Site-Resolved Observation of Vibrational Energy Transfer Using a Genetically Encoded Ultrafast Heater. *Angew. Chemie - Int. Ed.* doi:10.1002/anie.201812995.

Exner, M. P., Kuenzl, T., To, T. M. T., Ouyang, Z., Schwagerus, S., Hoesl, M. G., et al. (2017). Design of S-Allylcysteine in Situ Production and Incorporation Based on a Novel Pyrrolysyl-tRNA Synthetase Variant. *ChemBioChem* 18, 85–90. doi:10.1002/cbic.201600537.

Owens, A. E., Grasso, K. T., Ziegler, C. A., and Fasan, R. (2017). Two-Tier Screening Platform for Directed Evolution of Aminoacyl–tRNA Synthetases with Enhanced Stop Codon Suppression Efficiency. *ChemBioChem* 18, 1109–1116. doi:10.1002/cbic.201700039.

Pott, M., Schmidt, M. J., and Summerer, D. (2014). Evolved sequence contexts for highly efficient amber suppression with noncanonical amino acids. *ACS Chem. Biol.* 9, 2815–2822. doi:10.1021/cb5006273.
